# Timing and location of reproduction in African waterfowl: an overview of >100 years of nest records

**DOI:** 10.1002/ece3.1853

**Published:** 2016-01-18

**Authors:** Graeme S. Cumming, Douglas M. Harebottle, Josphine Mundava, Nickson Otieno, Stephanie J. Tyler

**Affiliations:** ^1^ Percy FitzPatrick InstituteDST/NRF Centre of ExcellenceUniversity of Cape TownRondeboschCape Town7701South Africa; ^2^ Animal Demography UnitDepartment of Biological SciencesUniversity of Cape TownRondebosch7701South Africa; ^3^ National University of Science and TechnologyP.O Box AC939AscotBulawayoZimbabwe; ^4^ Ornithology DepartmentNational Museums of Kenya40658NairobiKenya; ^5^ Yew Tree CottageLone LanePenalltMonmouthshireWalesNP25 4AJU.K; ^6^ BirdLife BotswanaGaboroneBotswana; ^7^ ARC Centre of Excellence for Coral Reef StudiesJames Cook UniversityTownsvilleQueensland4811Australia; ^8^ Department of Biodiversity and Conservation BiologyUniversity of the Western CapeP/Bag X17Bellville7535South Africa

**Keywords:** Anatidae, Botswana, breeding, Kenya, mortality, nesting, predation, reproduction, South Africa, waterfowl, Zimbabwe

## Abstract

The timing and location of reproduction are fundamental elements of reproductive success for all organisms. Understanding why animals choose to reproduce at particular times and in particular places is also important for our understanding of other aspects of organismal ecology, such as their habitat requirements, movement strategies, and biogeography. Although breeding patterns in waterfowl are relatively well documented, most studies are from northern temperate regions and the influences of location and time of year on breeding in Afrotropical ducks (Anatidae) are poorly understood. We outline six alternative (but not mutually exclusive) hypotheses that might explain where and when Afrotropical ducks choose to breed. To explore these hypotheses, we assembled and analyzed a new database of *c*. 22,000 breeding records for 16 Afrotropical ducks and one introduced Palearctic species (the Mallard *Anas platyrhynchos*). The full database is available on line as an appendix to this article. We identified five distinct breeding strategies as well as two outliers. Peak breeding for 9 of 16 indigenous duck species occurs during the dry season. We found no evidence for spatial synchrony or spatial autocorrelation in breeding, suggesting a high level of flexibility in waterfowl responses to prevailing conditions in any given year. More intensive analyses of alternative hypotheses are needed, but our initial analysis suggests that the timing of breeding for the majority of Afrotropical ducks is driven by a combination of resource availability and predation risk.

## Introduction

The timing and location of reproduction are central elements of the life history strategy of any organism. While short‐lived organisms that produce large numbers of small offspring are under heavy selective pressure to reproduce at a time of year and in a location that is favorable for juvenile survival, longer‐lived organisms face trade‐offs between their own survival and that of their offspring as well as between offspring quality and offspring quantity (Sibly et al. 2012). The Anseriformes (swans, geese, and ducks) are relatively well‐studied, but the trade‐offs that they face in selecting a time and location for reproduction are poorly understood. The gaps in our current understanding are particularly apparent in the case of Afrotropical ducks, which appear to exhibit a wide range of breeding strategies. Although these differences have been attributed to differences in their foraging styles and responses to rainfall (Little et al. 1995), there is a wide range of plausible hypotheses that might explain the breeding patterns of Afrotropical ducks. These hypotheses have not previously been tested, or even rigorously described, in a quantitative framework.

All Anseriformes produce precocial young and investment in eggs is relatively high for their body size (Sibly et al. 2012). Egg production demands a substantial investment of lipids, often but not always from internal nutrient reserves (Ankney et al. 1991; Alisauskas and Ankney 1994; Hobson et al. 2004). The juveniles of most duck species cannot fly until they are at least 8 weeks old (Lee and Kruse 1973; Milstein 1993; Hockey et al. 2005), demanding a further investment in parental care, suitable proximity of nesting sites to waterbodies that will not dry down during the nesting period, and potentially increased exposure of adults to both terrestrial and aerial predators. Adults of some African ducks, such as the Egyptian Goose *Alopochen aegyptiacus* (a shelduck, not a true goose), also expend considerable effort finding and defending nest sites and breeding territories (Milstein 1993). Most Afrotropical ducks lay only one or two clutches of eggs per year (Milstein 1993; Hockey et al. 2005) and are highly mobile (Cumming et al. 2012a), meaning that they have a wide range of possible breeding locations and times from which to select.

Given the reliance of waterfowl on water and water‐associated resources, it might be expected that they would choose to breed at times and in places when water is at a maximum. Water and food availability for ducks are not necessarily synchronous, however; depending on the needs and growth rates of plant and macroinvertebrate populations, and the relationships between water depth and food availability for ducks, foraging conditions may be better some months after peak water availability than during the peak itself (Cumming et al. 2012b). Organisms that are strongly conditioned by their environment are also expected to show a high degree of spatial synchrony in reproductive patterns and predictable shifts in breeding times with latitude (Sæther et al. 2008). Most waterfowl research has been undertaken in northern temperate regions, where ducks breed synchronously in spring at a time when food and water are plentiful and temperatures are warm. In North America, for example, estimates of available wetland area in the prairies in May, together with aerial population surveys, provide indicators of population‐level production that are sufficiently reliable to be used to set hunting quotas (Johnson and Grier 1988; Klett et al. 1988; Nichols et al. 1995). With a short breeding season, flightless molt must occur prior to migration and individuals have little choice in breeding time. The constraints of temperate seasonality therefore make it difficult to differentiate between alternative drivers of life history strategies. In sub‐Saharan Africa, by contrast, two important constraints on breeding are removed: winters are mild, and birds do not undertake regular south–north migrations.

There at least six alternative hypotheses that might explain the decisions that are made by African ducks about when and where to breed (Table 1). It is not possible to contrast the hypotheses in Table 1 rigorously without introducing a wide range of other supporting data and analyses, but the obvious starting point for teasing these hypotheses apart is to document and map existing patterns in available breeding data. Our goals in this paper were thus (1) to clarify and quantitatively describe the breeding patterns of ducks, based on the best available information; and (2) to summarize and provide a preliminary evaluation of competing hypotheses that might explain the timing and location of breeding. These steps are intended to provide the groundwork for further, more intensive analyses of individual hypotheses rather than to offer a final solution.

**Table 1:** Alternative hypotheses that might explain when and where African waterfowl reproduce

Hypothesis	Explanation and assumptions	Expected time of reproduction	Comments
Juvenile food availability	Timing and location driven by resources (food and water) available for juveniles	Uncertain	Potential differences between duck foraging styles (diving, dabbling, or grazing); timing of peak resources for juveniles currently impossible to quantify due to lack of dietary and hydrological data
Protein limitation	Afrotropical waterfowl that breed in temporary waterbodies may be protein‐limited due to the relatively low availability of macroinvertebrates, hence dependent on production of nitrogen‐rich *Panicum* grass species (Petrie 1996)	Spring/summer (rainy season)	Possible conflict with other evidence (Hart 1985) suggesting most abundant aquatic invertebrates in mid‐winter
Predation	Adults breed when the risk of predation on adults and/or juveniles is low	Winter (dry season)	Breeding when resources are at a peak does not disprove this hypothesis; breeding when resources are off‐peak would disprove food availability hypothesis
Overheating when brooding	Breeding during colder times of year may be favored to reduce the problem of overheating while brooding (Gillis et al. 2012; Cadena 2014)	Winter (dry season) or cooler summer months in highveld locations	Ducks have very dense, waterproof plumage and cannot sweat; many use heat exchange through their legs to thermoregulate
Flood risk to nests	Birds that breed near to seasonal wetlands may do so when wetlands are drying down, to avoid the risk of having the nest flooded during brooding	Late summer/early winter	Not relevant for tree ducks
Molt domination	The timing of flightless molt may dominate life history strategies, with birds timing reproduction secondarily to the optimal molting period and location	Variable with species	Most likely to be relevant for species that undertake molt migrations and have highly synchronized molt periods

We first assembled all available nest record data for southern and eastern Africa. We then asked three sets of fundamental ecological questions: (set 1) what patterns exist in the timing of breeding of African ducks, can we group duck species by shared strategies in the timing of breeding, and do the majority of species breed during resource‐rich times of year? (set 2) Do clear regional differences occur in the breeding times of different populations of African duck species? (set 3) Do species with larger ranges also show greater variability in the timing of breeding? The answers to these questions have important implications for our understanding of the timing and location of reproduction in waterfowl and offer a starting point for more intensive analyses of the alternative mechanisms proposed in Table 1.

## Methods

### Data sources

In many African countries, and particularly those that were once British colonies, groups of enthusiasts comprising mainly amateur ornithologists have for many years assembled natural history data about birds. Many local bird clubs across southern and eastern Africa used to run schemes to print, collect, and archive cards on which club members recorded the details of nests seen by chance or as they went birding. These cards were collected without any formal sampling design and many were stored only in hard copy form. Most have gradually made their way into a small set of national repositories, either at national offices of BirdLife International (see http://www.birdlife.org/), at museums, or at universities. The successors of bird card initiatives have been geographically extensive bird atlases, with sampling designs created by professional biologists and statisticians but actual sampling largely undertaken by amateurs. Despite the quantities of data available, there have been very few scientific analyses of sub‐Saharan African nest record data for waterbirds (Little et al. 1995; Hockey et al. 2005).

The data in this paper include all available nest record data sets for Afrotropical ducks from the southern and east African regions, with representation from national databases (Appendix S1) that are currently held and maintained in seven African countries (Botswana, Kenya, Namibia, South Africa, Tanzania, Zambia, and Zimbabwe). Records are included from another four countries (Angola, Burundi, Mozambique, Rwanda) that lack their own nest record schemes. This analysis is thus of unprecedented geographic scope and detail.

Many nest records captured on cards were digitized for the first time for this project. For Botswana, South Africa, Namibia, and Zimbabwe, we included additional digital records from bird atlasing efforts (e.g., Harrison et al. 1997) that were not part of the national bird card data set. For Botswana, records published in “The Babbler*”* (the biannual newsletter of what was the Botswana Bird Club, now BirdLife Botswana) had not been digitally captured and were entered separately. We were also given access to several additional data sets, including a long time series of breeding observations from Rocher Pan in South Africa.

### Data capture and processing

Nest record data were captured (Appendix S1) and then re‐checked row by row for accuracy. A summary of data capture considerations is given in Appendix S2. We georegistered as many of the records as possible. Some were associated with four‐ or six‐letter grid references (corresponding to 15′ × 15′ grid cells) when collected; others gave only a place name and region. Coordinates were tracked down using the Google search engine and Google Earth. We assigned locations to the nearest plausible point that reflected the ecology of the duck species concerned. Grid references were assigned to the grid cell's center to minimize possible error. The potential accuracy of each record was estimated and we assigned an error code to each record (Appendix S2). Where clear identification of location was not possible, records were excluded, as were those with ambiguous data, such as missing dates or indecipherable species names. We estimated hatching date for all records to assign them to a standard point in the breeding cycle. “Breeding time” thus refers to hatching date unless otherwise specified. Further details on hatching date estimation are given in Appendix S2.

### Data summary

The final data set (Appendix S3) contained 22,057 records of suitable quality (i.e., both hatching date and location could be reasonably estimated). The data included records for all 16 of the common duck species that are considered Afrotropical in origin, as well as 11 records for one introduced Palearctic species, the Mallard *Anas platyrhynchos* (Table 2). Although this sample size is small, we included the data because they provide some interesting insights. Records were unevenly distributed across indigenous species, ranging from 6878 records for Egyptian Goose to 77 records for Hottentot Teal *Anas hottentota*. No records were available from Madagascar, and so Madagascan endemics (e.g., Madagascar Teal, *Anas bernieri*) were excluded; and although a few breeding records exist from Angola for Hartlaub's Duck *Pteronetta hartlaubi*, the locations of these observations could not be pinpointed with sufficient accuracy to include them.

**Table 2:** Numbers of breeding records for each species included in the final database

Duck Species	Common Name	Foraging style	Records
*Alopochen aegyptiaca*	Egyptian Goose	Grazing	6877
*Anas capensis*	Cape Teal	Dabbling	2163
*Anas erythrorhyncha*	Red‐billed Teal	Dabbling	768
*Anas hottentota*	Hottentot Teal	Dabbling	77
*Anas platyrhynchos*	Mallard	Dabbling	11
*Anas smithii*	Cape Shoveler	Dabbling/filter feeding	4117
*Anas sparsa*	African Black Duck	Dabbling	339
*Anas undulata*	Yellow‐billed Duck	Dabbling	3561
*Dendrocygna bicolor*	Fulvous Whistling Duck	Dabbling	174
*Dendrocygna viduata*	White‐faced Whistling Duck	Dabbling	1006
*Netta erythrophthalma*	Southern Pochard	Diving	429
*Nettapus auritus*	Pygmy Goose	Dabbling	109
*Oxyura maccoa*	Maccoa Duck	Diving	372
*Plectropterus gambensis*	Spur‐winged Goose	Grazing	604
*Sarkidiornis melanotos*	Knob‐billed Duck	Dabbling	235
*Tadorna cana*	South African Shelduck	Grazing/Dabbling	681
*Thalassornis leuconotus*	White‐backed Duck	Dabbling	534
Total			22,057

**Figure 1:**
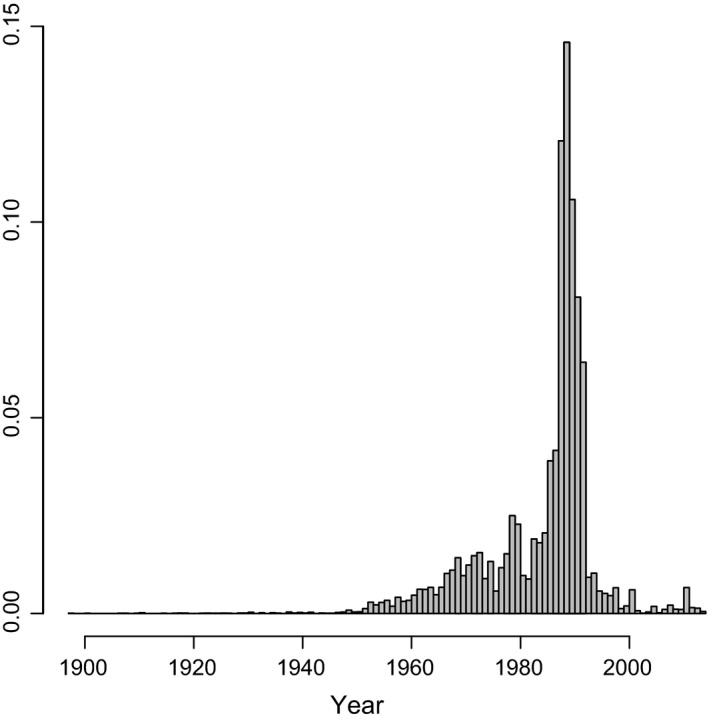
Histogram showing the proportion of bird nest records collected in each year from 1897 to 2014.

**Figure 2:**
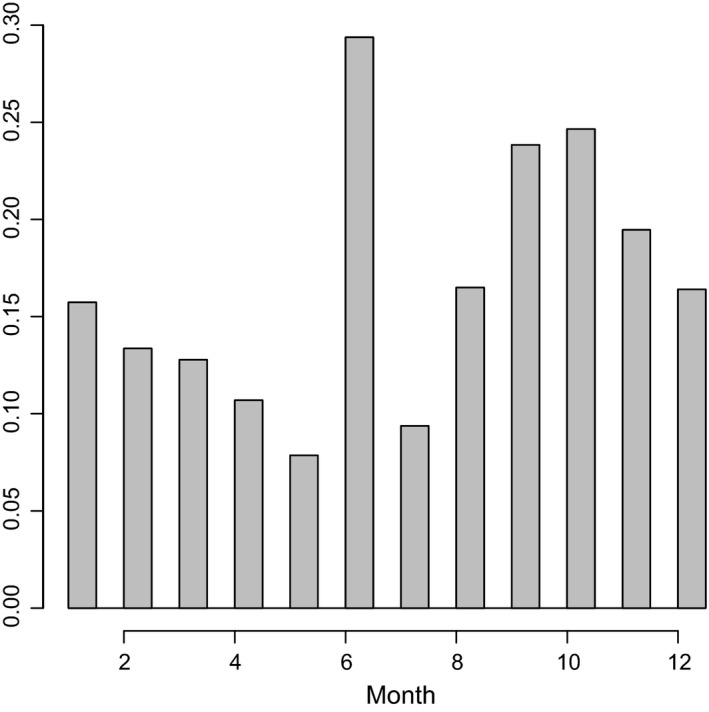
Histogram showing the proportional distribution of nest records by month, January (1) to December (12).

Records covered the time period 1897–2013, with a median value of 1987 and a mean of 1984 (Fig. 1). The 1970s and 1980s appear to have been the golden age of nest record returns by amateur ornithologists, although the impact of the first Southern African Bird Atlasing Project (SABAP1, duration approximately 1987–1991) is clearly visible in Figure 1. The observation frequency of breeding ducks was highest in June (Fig. 2), presumably reflecting the commonest breeding period for the most easily seen ducks. June is the middle of winter in the Southern Hemisphere and would generally not be considered a peak birding period, suggesting that the data set is sufficiently large for the detection of genuine trends.

**Figure 3:**
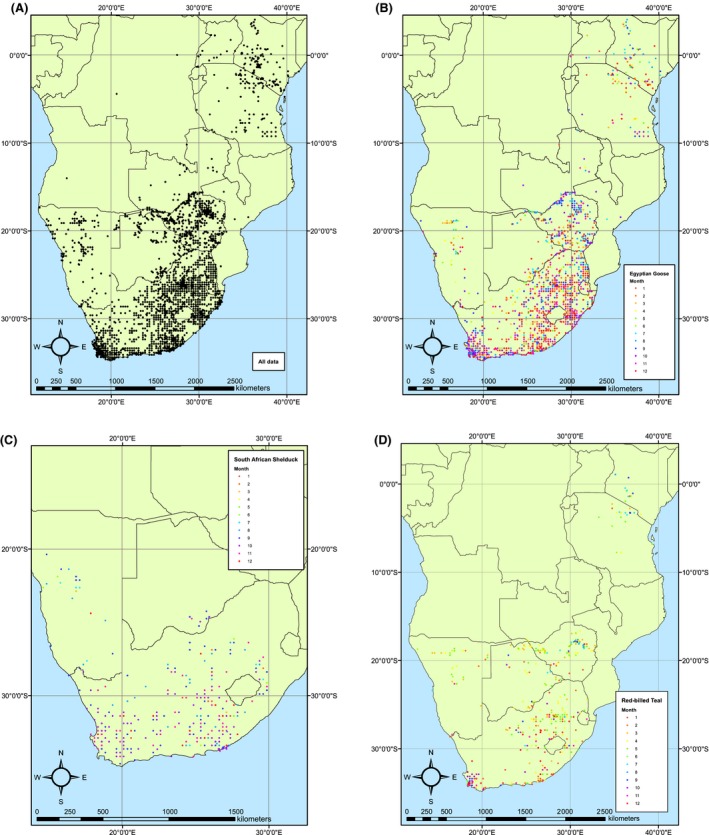
Maps of southern and east Africa, showing (A) locations of all breeding records used in the analysis; (B) an example data set showing breeding months and locations for the most‐recorded species, the widespread and apparently asynchronous Egyptian Goose *Alopochen aegyptiaca*; (C) a second example for a more localized and relatively synchronized breeder, the South African Shelduck *Tadorna cana*; and (D) a third example, a widespread mid‐ to late summer breeder, the Red‐billed Teal *Anas erythrorhyncha*. See Appendix S4 for all distribution maps.

Spatial coverage was variable, with highest observation densities near to large towns (particularly Cape Town and Johannesburg) and areas in which the birding community has been active for long periods of time (Fig. 3). When considered by country, 81% of records were from South Africa (*n* = 17,930), with substantial contributions from Zimbabwe (2178), Botswana (704), Namibia (596), and Kenya (400). At the other end of the spectrum, only three records were available from a single location in Angola, two from Mozambique, and two from the Democratic Republic of the Congo. Maps of all breeding locations for all species concurrently and for each species individually are presented in Appendix S4.

### Statistical considerations and analysis

The strengths of the data include their long duration, extensive coverage, and large sample size (with some variation between species). The weaknesses include the lack of a standardized sampling approach, the potential for errors (ranging from misidentifications to data capture errors), and the uneven coverage of records in both space and time. All analyses were run in R (R Development Core Team 2014). The analysis was structured to address each of our three focal sets of questions and is presented accordingly.

1. What patterns exist in the timing of breeding of Afrotropical ducks, can we group duck species by shared reproductive strategies, and do the majority of species breed during resource‐rich times of year?

We tested for general patterns in the timing of breeding (specifically, in hatching dates) by looking at trends by month across the full spatial extent of the data, ignoring differences in location. We quantified the timing of breeding by determining the number of all records that occurred within a given month and converting it to a proportion of the total number of breeding records for that species. This yielded a table of species (rows) and months (columns) in which each entry was a proportion. We tested for commonalities in general breeding patterns by clustering data according to the monthly values. As the data were time series data, and hence autocorrelated in time, we used the “tsclust” package in R, with the least squares distance option, to run a clustering algorithm that has been developed explicitly for analysis of time series (Manso and Vilar 2014). We tested the significance of clusters using the mrpp permutation test function in the “vegan” package of R (Oksanen et al. 2013).

To extend this analysis, we overlaid the coordinates of each breeding observation on an interpolated rainfall map taken from the CRES (Centre for Resources and Environmental Studies) database (Hutchinson et al. 1995). This data set consists of 60‐year mean (1920–1980) interpolated estimates on a monthly basis for rainfall from 6051 weather stations at a resolution of 0.05 degrees. Although more recent rainfall data are available, these data were the best fit that we could find to the time period of the majority of nest record observations. We extracted the mean rainfall for the recorded breeding month for each record and compared it to the mean annual rainfall at that location to determine whether each species was typically breeding during periods of above‐ or below‐average rainfall for their breeding location.

Do clear regional differences occur in the breeding times of different populations of any Afrotropical duck species?

This question is more easily answered for less mobile organisms. For example, breeding periods for large herbivore populations can be easily compared between protected areas that are geographically distinct (e.g., Moe et al. 2007). The problem is, however, far more complex for species that are highly mobile, unconfined, and potentially nomadic. The boundaries of individual populations of *Afrotropical* waterfowl are unknown, and some individuals have been shown to move over a thousand kilometers between molting and breeding sites (Underhill et al. 1999; Cumming et al. 2012a). Nor, based on other observations and preliminary analysis, can we simplify the problem by assuming that breeding correlates directly with rainfall or other abiotic features of the environment.

The seasonality of rainfall shows considerable variation over southern and eastern Africa; the study region includes 14 different Koppen–Geiger climate zones. To test for spatial differences in the timing of breeding in this data set thus required that we test for spatial autocorrelation (spatial synchrony) in breeding month. If there were spatial patterns in waterfowl breeding time, we would expect that breeding records be locally (spatially) clustered in time, with an increasing time difference between areas that are geographically further apart.

The breeding data are circular (i.e., month 1 and month 12 are more similar than month 1 and month 6). Statistical approaches for circular data generally require that data are either split into two angular components (a sin and a cosine function) or grouped spatially and converted to proportions. Running semivariograms with a bivariate response is problematic, and the nature of the data is such that sampling is irregular and patchy, making regular groupings (e.g., overlaying a half‐degree grid and undertaking analysis by proportion of records per grid cell) subject to strong sampling biases.

To solve these problems we used a simplified version of a correlogram that made sense for the peculiarities of our data set. Correlograms test for spatial autocorrelation under the assumption that the similarity between pairs of points will decline as points become further apart in geographic space (Isaaks and Srivastava 1989; Turner et al. 2001). The distance at which correlation drops away entirely (which is equivalent to the sill of the semivariogram) provides a measure of the scale at which the similarities or differences between pairs of individual records are independent of their location in geographic space.

We first converted our monthly data to degrees (month/12 * 360), visualizing each month as a point on a circle of unit radius. The length of the chord between two different months offers an indication of the separation of different months in time and has a more linear distribution than arc length. We then selected a pair of records at random from the data for a single species and calculated the chord length between them (“temporal distance”) and their geographic distance apart. Geographic distances were estimated from the original unprojected (latitude–longitude) coordinates using Haversine great circle distance in the “geosphere” package in R (Hijmans et al. 2012).

Plotting the geographic distance between pairs of points against their temporal distance should provide either a correlogram‐like curve (if nesting months are spatially autocorrelated) or a random scatter of points (if no autocorrelation exists). Some additional steps must however be undertaken to cope with (1) the potentially high variance in the data from overlapping populations; (2) the nonrandom sampling bias; and (3) the large sample size, for several species, which makes analysis of all possible pairs of points impractical. Even a data set of only 100 breeding records gives a potential 10,000 pairs of combinations, and fully inclusive analysis was not feasible with the nearly 7000 data points for Egyptian Geese.

We therefore ran the analysis species by species as follows: (1) select 10,000 pairs of points at random, with replacement; (2) calculate the geographic and temporal distances between each pair of points, in units of meters and radians, respectively; (3) use these “actual” data to estimate a mean and standard deviation for all distances; (4) repeat step 3 on a “null” data set in which the time values are randomly sorted, independent of the geographic distances, to break any spatiotemporal structure in the data; and (5) compare the correlations between geographic distances and temporal distances for both the “actual” and the “null” data sets, visually at first and then using Spearman's correlation coefficient, for each species. If there were spatial trends in the timing of breeding we would then expect to find a stronger correlation between temporal and geographic distances for the actual data than for the null data.

Do species with larger ranges also show greater variability in the timing of breeding?

To answer this question we combined the results for all species from the previous step and tested for trends, and for any sign of a significant slope, in either the means or the variances of the temporal distances as a function of their spatial differences. We ran a least squares regression analysis to test for a linear trend in the relationship between temporal distance and geographic distance across all 17 study species and used a *t*‐test on the coefficient value to determine whether the slope was significantly different from zero. To correct for possible sampling bias we also ran this analysis on a randomized “null” data set, with the expectation that if there were a significant trend in the actual data, the results would differ significantly from the null data. In other words, to accept the hypothesis that species with more extensive ranges also show significantly greater variability in the timing of breeding, we would need to find (when comparing between different species): (1) a significant, nonzero, increasing trend between mean temporal distance and mean geographic distance; (2) the lack of a trend, or at least a significantly lower slope, for the null data set; and/or (3) an increasing trend in the standard deviations of the temporal distances for each species, with either the magnitude of the standard deviation increasing with increasing range extent or one or more species showing nonoverlapping standard deviations.

## Results

The results of statistical analyses are best summarized under each of our three focal questions.

1. What patterns exist in the timing of breeding of African ducks, can we group duck species by shared reproductive strategies, and do the majority of species breed during resource‐rich times of year?

**Figure 4:**
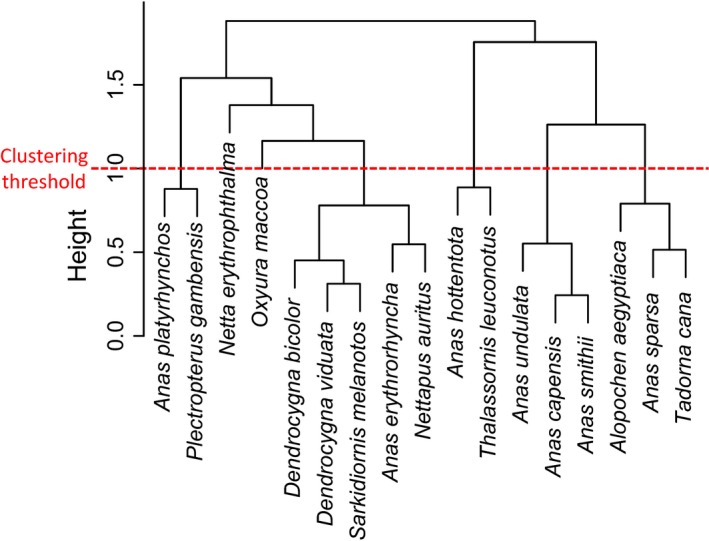
Results from time series clustering of proportional numbers of nest records by month for all species in the analysis. The red dotted line indicates the threshold value (1) that was used for grouping species into different life history syndromes. Common names for all species are given in Table 1.

**Figure 5:**
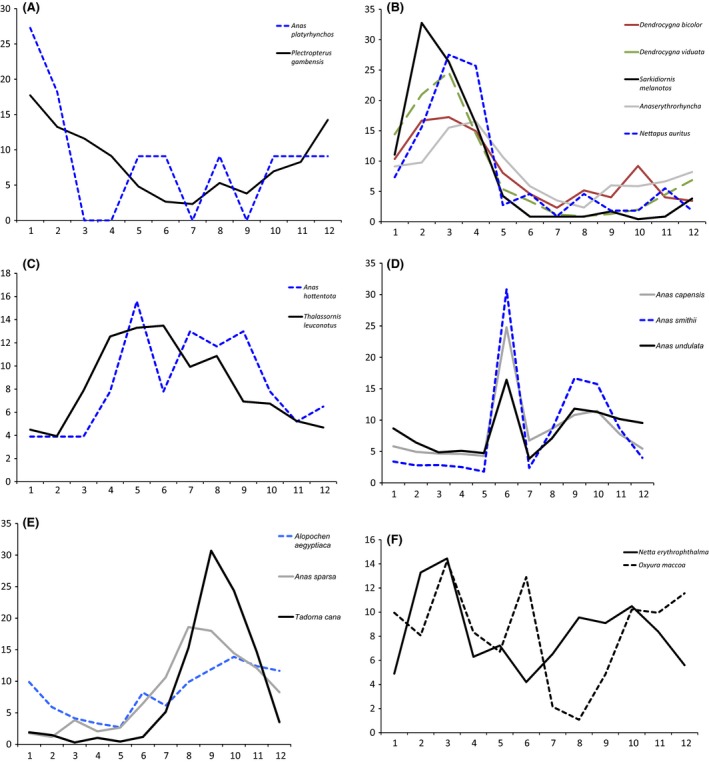
Regional summary of breeding patterns for all analyzed duck species. In each case the *y*‐axis indicates the percentage of all available records that occurred in a given month and the *x*‐axis indicates the month; the sum of all *y* values for any species is 100. The groups in each panel correspond to the clusters identified in Figure 4 and the text: (A) early summer/first rains breeders (Spur‐winged Goose, Mallard); (B) mid‐ to late summer/dry season breeders (Fulvous Whistling Duck, White‐faced Whistling Duck, Knob‐billed Duck, Red‐billed Teal, and Pygmy Goose); (C) multimodal but not mid‐summer breeders (Hottentot Teal, White‐backed Duck); (D) primarily mid‐winter breeders with a second peak in spring (Yellow‐billed Duck, Cape Teal, and Cape Shoveler); and (E) primarily late winter breeders, possibly with a smaller peak in mid‐winter (Egyptian Goose, South African Shelduck, African Black Duck). These syndromes exclude (F) the two diving ducks (Southern Pochard and Maccoa Duck), which appear to be multimodal and possibly aseasonal breeders.

Cluster analysis of time series of proportional records indicated that there were distinct reproductive patterns (Fig. 4). Permutation tests comparing within‐and between‐group differences suggested that a value of 1.17 offered a reasonable height at which to consider clusters within the dendrogram independent. Based on what is known about the foraging and breeding ecology of the different duck species (see also Fig. 5) we further separated the Maccoa Duck, which had a close‐to‐significant divergence height of 1.12 and quite different foraging ecology from the other species in the closest cluster. This divided the 17 species into five distinct clusters and two singletons. The five identifiable clusters were broadly characterized (Fig. 5) as (1) early summer/first rains breeders (Spur‐winged Goose, Mallard); (2) mid‐ to late summer/dry season breeders (Fulvous Whistling Duck, White‐faced Whistling Duck, Knob‐billed Duck, Red‐billed Teal, and Pygmy Goose); (3) multimodal but not mid‐summer breeders (Hottentot Teal, White‐backed Duck); (4) primarily mid‐winter breeders with a second peak in spring (Yellow‐billed Duck, Cape Teal, and Cape Shoveler); and (5) primarily late winter breeders, possibly with a smaller peak in mid‐winter (Egyptian Goose, South African Shelduck, African Black Duck). These clusters exclude the two diving ducks (Southern Pochard and Maccoa Duck), which appear to be multimodal and possibly aseasonal breeders. In Europe, Mallard are well‐documented summer breeders; as in New Zealand, they seem to have been able to adapt the timing of their breeding activities in southern Africa to fit Southern Hemisphere seasons.

There was considerable variation in the apparent level of synchrony in the timing of breeding within each breeding pattern cluster. As Figure 5 displays the timing of breeding records as a proportion of the total number of records, species with higher individual peaks and deeper individual troughs have greater synchrony, while those with lower peaks or higher troughs exhibit greater variability. For example, within Cluster 5 (late winter/early spring breeders), the South African Shelduck exhibits a high level of within‐population synchrony at the regional scale whereas the Egyptian Goose may potentially be found breeding in most locations at almost any time of year.

**Figure 6:**
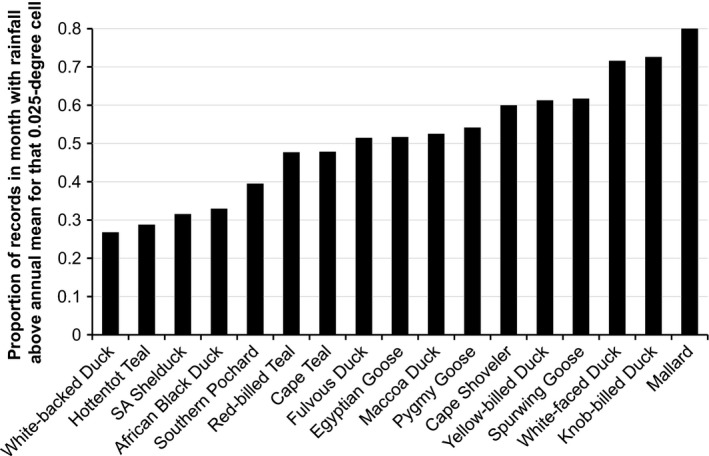
Plot of the proportion of nest records for each species from months with above‐average rainfall. These data were derived by comparing mean rainfall for the breeding month to mean rainfall across all months at the same location at 0.05‐degree resolution. Species to the left‐hand side of the figure breed in the drier half of the year; those in the middle (i.e., with values in the 50–60% range) appear to either breed during periods with some rainfall, but not at the wettest time of the year, or to show high variation (no pattern) in breeding time in relation to rainfall; and those toward the right hand side of the figure breed more frequently during the wetter months of the year.

Analysis of rainfall during the breeding month in relation to mean annual rainfall indicated that seven species (White‐backed Duck, Hottentot Teal, South African Shelduck, African Black Duck, Southern Pochard, Red‐billed Teal, and Cape Teal) consistently breed at a time of year in which rainfall is below the annual mean (Fig. 6). A further four species (Fulvous Duck, Egyptian Goose, Maccoa Duck, and Pygmy Goose) do not appear to routinely breed during wetter months, with only about 50% of breeding records coming from months with rainfall above the annual mean. Five species (Cape Shoveler, Yellow‐billed Duck, Spurwing Goose, White‐faced Whistling‐Duck and Knob‐Billed Duck) bred more frequently in months with above‐average rainfall, and nearly all records of breeding Mallard were from wetter months of the year.

The appearance of bimodality or multimodality from aggregated regional data can be deceptive if there are spatial differences in breeding times, such that populations in each individual locality breed only once a year. Rather than being genuinely bimodal, it may simply be (for example) that for a given species, populations living in the north of the study region breed in mid‐winter and those living in the south breed in spring. This observation leads on to our next question.

Do clear differences occur in the breeding times of different populations of any African duck species?

Visual comparison of temporal distance (chord length between months) and geographic distance (in km) for each species showed no clear trends and no indication of a sill or a range in any case. Spearman's correlation coefficient for the relationship between temporal and geographic distance was significant to *P* < 0.01 for all species (ρ between 0.04 and 0.25; *n* = 10,000) except one, the (introduced) Mallard, for which the sample size was inadequate to reach a strong conclusion. This might in theory be interpreted as showing that local spatial differences exist in the timing of breeding of all of our study species except the Mallard. However, further testing indicated that the outcome was a consequence of the biased sampling regime and the high statistical power afforded by a large sample size (*n* = 10,000) rather than any biologically meaningful relationship. The randomized data, in which the true relationships between geographic and temporal distance were destroyed, showed exactly the same pattern. When compared across all species in the analysis there was no difference in Spearman's correlation coefficient values between the randomized and the actual data (Wilcoxon signed ranks test *W* = 189, *P* < 0.89). These statistical conclusions are further supported by visual inspection of the distribution maps for breeding records for individual species (Appendix S2), which show a notable lack of clusters of points of a single color when breeding locations are shaded by month.

Do species with larger ranges also show greater variability in the timing of breeding?

**Figure 7:**
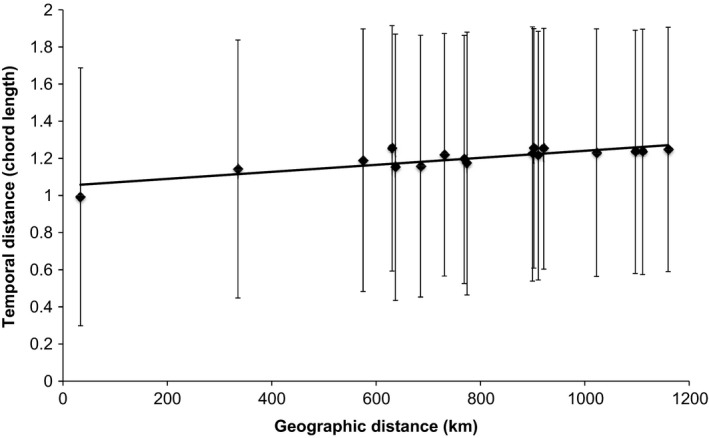
Plot of the mean and standard deviation in chord lengths between months, on the *y*‐axis, as a function of the separation of points in geographic space (*x*‐axis). Each point on this figure represents a different duck species. Although the slope of the line was significantly different from 0, its slope did not differ from that produced by a randomized null model and there were no outliers, indicating a lack of spatial synchrony in breeding patterns across all species. Further details in text.

Although there was no evidence of spatial synchrony for any of our study species, it was still possible that there were differences in the spatial variability of the timing of breeding between species. The relationship between temporal distance (chord length) and geographic distance (Fig. 7) for all 17 species in the analysis was significant (*r* ^2^ = 0.76, *P* < 0.005) and the slope of the line of best fit to the mean values was, although very shallow, significantly different from zero (*x* = 0.00019, *t* = 6.92, *P* < 0.001). However, the standard deviations around the data indicated that there were no significant differences between species in terms of variation in breeding times, and both the mean and the standard deviation of the actual data were nearly identical to those of the randomized data (Spearman's ρ > 0.98 in both cases, *P* < 0.001, *n* = 17). The slope of the line was also very shallow, even though nonzero. The correlation between geographic distance and temporal distance in these data therefore appeared to be a statistical artifact rather than an indication of any underlying ecological mechanism; ducks with larger ranges (higher mean geographic separation between 10,000 randomly selected pairs of breeding points) did not show significantly greater differences or variations in breeding time across their ranges.

## Discussion

Our results show clearly that at the regional level there are distinct breeding patterns in African waterfowl, with at least five different strategies being apparent. Intriguingly, however, we found no evidence at this scale of analysis for spatial autocorrelation or spatial synchrony in the timing of breeding, and no clear support for the hypothesis that species with larger ranges should also show greater variability in the timing of breeding. Our analysis also highlights some of the strengths and weaknesses of nest record data. Nest records have provided an essential starting point for understanding the nesting patterns of Afrotropical ducks and this paper and associated database provide a strong baseline for future research. Despite the large numbers of nest records that have been collected and their potential scientific value, however, the lack of a more standardized collection protocol has reduced the overall usefulness of the data set. Given recent declines in nest card returns we would advocate a restructuring of nest card collection protocols and a renewed investment in the collection of breeding data, including records of sampling events when no nests were seen and following the guidelines proposed for atlasing efforts by Robertson et al. (2010).

For 17 duck species to show evidence of five cohesive breeding patterns comes as no surprise. What makes less intuitive sense is that 9 of 16 indigenous species have peak hatching dates during winter (June and July) and the transitional period (August to October, depending on latitude) before the rains come, when surface water is declining, major crops have been harvested (with the exception of winter wheat), temporary pans are dry, and vegetation productivity is lower in all areas except the winter rainfall region in the southwestern Cape of South Africa (Unganai and Kogan 1998; Azzali and Menenti 2000; Nicholson 2000). Our analysis of local rainfall (Fig. 6) shows that only five indigenous species breed primarily in months that are wetter than average at their chosen breeding location.

In assessing the data it is important to note that there are relatively few catchments in our study areas in which long lags separate rainfall and water flows into lakes. South Africa, from which the majority of records come, is generally arid and has relatively short, high gradient river systems and few floodplains or deltas; many of the natural areas in which ducks reproduce are dominated by shallow, seasonal pans that fill with the rains and dry down rapidly in the winter, rather than by the deeper lakes that dominate Northern Hemisphere systems. The most obvious exception to this general rule is the Okavango system and particularly Lake Ngami, which receives dry season flows from the Angolan highlands, but the proportion of nest records from this region is relatively small. In floodplain systems, such as in the mid‐Okavango River, duck species follow a successional pattern driven by the timing of peak flow, and depending on foraging style: diving duck numbers peak first, followed by dabbling and then grazing ducks (Cumming et al. 2012b).

Returning to the hypotheses in Table 1, the most obvious likely driver of reproduction is juvenile food availability (Cumming and Bernard 1997). It is possible that aquatic invertebrate densities and/or numbers of aquatic plant seeds are higher in permanent or drying‐down water bodies in mid‐winter (Hart 1985). This will not, of course, be the case in smaller waterbodies that have d ried up. Southern Africa in particular has relatively few large waterbodies but there is a likely trade‐off through the dry season between food density and the total area of available foraging habitat, with food density in drying‐down waterbodies increasing as other waterbodies go fully dry, and optimal strategies may vary spatially. The adults of most of our study species are predominantly plant feeders (Milstein 1993; Petrie 1996, 1997, 2005; Hockey et al. 2005), with some exceptions (e.g., Cape Teal have been recorded eating 83% animal matter, and Cape Shoveler 70%; Hockey et al. 2005). The diets of juvenile ducks may, however, differ substantially from those of their parents. In the USA, juvenile Ring‐necked Duck ate “mostly invertebrates,” shifting to plant matter as they grew older (Hohman 1985). Juvenile Black Duck in Maine consumed 88–91% invertebrate matter by dry mass when partially feathered, decreasing to 43% for “fully feathered young” (Reinecke 1979). In Northern Maine, 23 sampled juvenile pintails consumed a diet of 66% “animal foods”; this proportion rose to 81% for eight flightless juveniles, while nonbreeding adults consumed roughly equal proportions of animal and plant matter (Krapu and Swanson 1977). Juvenile Wood Duck in Tennessee, by contrast, had 87% plant matter in their diets (Hocutt and Dimmick 1971).

Very little is known about the diets of the juveniles of most African species. According to Hockey et al. 2004, “half‐grown ducklings” of the White‐faced Duck consumed 93% plant matter; ducklings of the White‐backed Duck had “large quantities of seeds” in their gizzards, as well as Chironomid larvae in their stomachs; and South African Shelduck juveniles “feed largely on submerged aquatic vegetation, including algae.” By contrast, prefledging juvenile Yellow‐billed Duck have been documented to eat only 29% plant matter, increasing this amount to 83% as adults. Drying wetlands may host proportionally higher densities of invertebrates and drying may trigger the release of seeds of wetland‐adapted plants, possibly resulting in greater food availability for juvenile ducks. Given the high variance in water availability, wetland resources and drydown times across southern and eastern Africa, and the confounding influence of managed dams, it is extremely difficult in the absence of further research to determine whether Afrotropical ducks time reproduction to match the dietary needs off their offspring. This question could be resolved by detailed analysis comparing juvenile diets across different duck species under natural conditions, paired with sampling that focuses on quantifying the relative abundance of the same food items across a range of wetlands of different types (e.g., natural seasonal, natural permanent, riverine, and managed impoundment), possibly paired with stable isotope analysis of feathers to determine protein content in the diet.

Nests of ground‐nesting ducks have been shown to be more likely to succeed when vegetation cover around wetlands is thicker (Schranck 1972); the middle and end of the dry season are times when over most of the region, the opposite is true (Gaidet et al. 2012). Cape Teal, Cape Shoveler, and Yellow‐billed Duck, for example, all breed on wetland margins and exhibit peak breeding around the middle of winter (June). At typical southern African wetlands, where trampling by game or cattle commonly creates a “picosphere” of bare ground around wetlands in the dry season, sites where duck nests will not be more exposed in the dry season will be less common anywhere outside the winter rainfall region. Egyptian Geese at Lake Chivero near Harare (Zimbabwe), for example, typically breed on reed‐covered islands or in trees between July and September when grazing is at its poorest and water levels are low. Also of interest is that the few existing records for introduced Mallards suggest that these birds breed in the middle of summer (January in the Southern Hemisphere), as in the Northern Hemisphere, rather than in the middle of the Northern Hemisphere summer (June).

Dry season breeding may occur to minimize the risk of flooding of nests (Shine and Brown 2008), but this hypothesis seems unlikely as a general explanation for winter breeders given that several species nest in trees. Hockey et al. (2004) have suggested that South African Shelduck may preferentially nest in holes in the ground in order to reduce the challenges of thermoregulation while breeding in arid environments, but the hypothesis that adult ducks breed in winter to avoid overheating while brooding (Gillis et al. 2012; Cadena 2014) seems unlikely given that several duck species (including the Spurwing Goose, the largest African duck species and hence the species with the smallest surface area to volume ratio) breed successfully in the middle of summer. We do not, however, have any direct data on breeding success against which to validate this claim.

Predation is difficult to quantify but seems very likely to play an important role in waterfowl survivorship. While it may seem counter‐intuitive to breed in or near to a wetland at a time of year when predators are likely to be short of food, few avian or mammalian predators in southern and East Africa have offspring during the dry season (Skinner and Smithers 1990; Hockey et al. 2005), and hence the total energetic demands associated with a single predator territory may be lower. In addition, the clearer, more open shorelines of partially dried wetlands will reduce cover for predators and although they may make it harder for nests to remain undetected, this is less relevant for tree‐nesting ducks; open shorelines will also make it easier for adults and older ducklings to detect approaching predators and swim to safety.

Studies from other continents suggest that predation on duck nests is a major source of mortality (Pasitschniak‐Arts and Messier 1996; Pasitschniak‐Arts et al. 1998; Phillips et al. 2003). There are numerous accounts of juvenile ducks being taken by African Fish Eagles and Wahlberg's Eagles (Hockey et al. 2004) and observations at Barberspan, South Africa, also suggest that jackal predation is a major cause of mortality of Egyptian Geese and Yellow‐billed Ducks (Cumming pers. obs. and Cumming and Ndlovu 2011). Unpublished observations from Rocher Pan indicate high egg and nestling mortality from water mongooses *Atilax paludinosus* (K. Shaw, pers. comm.). Survivorship and predation data for Afrotropical waterfowl collected using standardized study protocols are however scarce, and we do not currently have the data with which to run a definitive test of this hypothesis.

We therefore interpret our results as suggesting that the interactions of food availability and predation are the most likely drivers of the timing of breeding for the majority of waterfowl in sub‐Saharan Africa. This is not a novel hypothesis; Geldenhuys (1980) argued that SA Shelduck breed in the dry season because (1) submerged aquatic plants are readily available to the young; and (2) littoral vegetation is sparse, aiding in predator detection. Further research on juvenile diets and the phenology of food and water availability to juveniles and breeding adult ducks in African wetlands, and better documentation of conditions for nest success, predation, and predation impacts on duck populations in Africa, appear to be critical for our further understanding of their reproduction, molt, and movement patterns.

The lack of local spatial autocorrelation in breeding times in our data was also unexpected, given that clear gradients in temperature and rainfall exist across the region (Nicholson 2000; Tyson et al. 2002). Our results might suggest that (1) ducks are highly conservative, with breeding occurring at the same time of year regardless of location; (2) ducks are highly flexible, with responses to local conditions dominating the choice of when to breed; or (3) that the temporal resolution of the data is too coarse (months rather than days) to detect trends. The third possibility seems unlikely given the large sample sizes for some species and the likelihood of random errors. Given what we know about variability in breeding times for some species (e.g., Egyptian Goose and Red‐billed Teal), the high interannual variation in precipitation and related water levels across southern Africa, and the high mobility of our study species, the second hypothesis seems most plausible. We would therefore argue that although ducks have evolved a set of distinct breeding patterns at a regional scale, reproduction is flexible and opportunistic. Such plasticity would be consistent with surviving in both an environment in which resources are highly variable and a high predation environment, given that choice of breeding site and timing of breeding can be viewed as a form of inducible defense (Cressler et al. 2010).

As indicated in Table 1, our results must also be considered in the context of the relationships between breeding and molt. All ducks undergo flightless molt once a year and during this period, birds are at potentially high risk of predation while they regrow their wing feathers. As the timing of flightless molt exhibits some flexibility in captive Afrotropical waterfowl, and energetic demands are not excessive (Ndlovu et al. 2010; Ndlovu 2012), we consider it unlikely that the relative timings of flightless molt and reproduction are tightly coupled. However, we would expect that flightless molt, which is a high predation risk period (Portugal et al. 2010), would be highly synchronized within different populations and should coincide with a period (and habitat) of low predation risk. Available evidence supports this prediction; the timing of molt is heavily synchronized and highly predictable within local populations for most Afrotropical duck species (Hockey et al. 2005). Flightless molt is usually undertaken in large groups that provide an effective predator detection and early warning system (Schmutz et al. 1983; Tamisier 1985; Cresswell 1994). Satellite‐tracked Egyptian Geese show a high degree of molt site fidelity, returning over a thousand kilometers in some cases to molt at a secure wetland before returning to their usual foraging and breeding area (Cumming et al. 2012a). Flightless molt in African waterfowl thus appears to be everything that reproduction is not: predictable, gregarious, highly synchronized for different populations in both space and time, and strongly influenced by site fidelity.

Nonconsumptive impacts of predators on reproductive parameters have been shown for a variety of bird species (Thomson et al. 2006; Cresswell 2008; Hua et al. 2014). The focus of most previous research on the impacts of predation on life history strategies has been on the trade‐offs between fecundity and survival, with correlated changes in parental investment strategies being largely ignored (Ghalambor and Martin 2000; Hua et al. 2014). Predation can impose both a survival cost through predation on the adult and a fecundity cost through predation on the offspring (Magnhagen 1991; Hua et al. 2014). In African habitats these trade‐offs must be seen through the lens of the need to breed in highly variable habitats that have suitable water and food resources for both brooding adults and juveniles. Although we currently lack the data with which to directly explore such trade‐offs, our results suggest that a better understanding of (1) juvenile food demands in relation to hydrological parameters, and (2) predation and its influences, will be critical to understanding the current life history strategies of Afrotropical ducks and the evolution of their lifestyles.

## Conflict of Interest

None declared.

## Supporting information


**Appendix S1.** Data sources. This appendix details the data sources that we used to create the database used in this analysis.

**Appendix S2.** Data capture considerations. This appendix contains a summary of the approach that we used in data capture to ensure consistency in data capture, coordinates, and estimates of hatching date.

**Appendix S3.** The database of duck breeding data that we used for our analysis. Details of source databases and data capture considerations are given in Appendices S1 and S2. These data are freely available for general use, with the one requirement that this paper should be cited as the data source whenever the data are used or presented. Any additional questions should be addressed to the corresponding author and/or the listed “owner” of the data set.

**Appendix S4.** Distribution maps of breeding data for all Afrotropical species considered in the analysis.
