# Supplementary material for: Timing and location of reproduction in African waterfowl: an overview of >100 years of nest records
Source: Ecol Evol. 2016 Jan 18;6(3):631–46. doi: 10.1002/ece3.1853 (PMC4739573; doi:10.1002/ece3.1853)
Supplement: Supplementary file 1 — Appendix S1. Data sources. This appendix details the data sources that we used to create the database used in this analysis. [file ECE3-6-631-s001.docx]

**Summary of Nest Card Data Sources**

Note that this list excludes countries, such as Rwanda and Mozambique, that feature in the data set but do not have their own nest card scheme. The holdings of the Nairobi Museum cover East Africa in general, rather than only Kenya; and the SAOS and SABAP 1 data sets from South Africa similarly include some data from other countries.

| Country | Data Sources | Location of holdings | Details of collection | Current contact details for individuals |
| --- | --- | --- | --- | --- |
| Botswana | BirdLife Botswana (BLB) nest record cards and data in BLB’s journal *Babbler*; additional data from Ken Oake | BirdLife Botswana offices in Maun and Gaborone | Bird nest card data collection was started under the auspices of the Botswana society and continued with the formation of the Botswana Bird Club (BBC) in 1980. The BBC became BirdLife Botswana in 2000. Dr. Neville Skinner analyzed data from the cards up to the mid-1990s in a series of three papers published in *Babbler;* further data collected by Birdlife Botswana have been published annually by Stephanie Tyler and Chris Brewster in *Babbler* issues*.* There are about 3,000 nest cards for waterbirds. | BirdLife Botswana Office in Gaborone. email: [blb@birdlifebotswana.org.bw](mailto:blb@birdlifebotswana.org.bw)  Dr Stephanie Tyler [steph_tyler2001@hotmail.com](mailto:steph_tyler2001@hotmail.com)  Chris Brewster [cbandog@gmail.com](mailto:cbandog@gmail.com) |
| Kenya | East Africa Nest Record Card Scheme, National Museums of Kenya Bird Skin Specimen Cards, Nature Kenya Wednesday Morning Birdwalk records | Ornithology Section, National Museums of Kenya, Nairobi | The collection of bird nest record cards at the National Museums of Kenya in Nairobi, is part of a bequest of natural history records and files from the East African Natural History Society, EANHS (now known as *Nature* Kenya). It was established in 1901 as a combined association of professional and amateur natural history enthusiasts in Kenya and Uganda. In 1978, the Ornithological Records Sub-committee created the East African Bird Card Records Scheme. It soon ran into challenges with record submission and in the mid-1980s, the East Africa Natural History Society handed the nest record cards to the Ornithology Department of the National Museums of Kenya, which has the national mandate for Ornithological research, collection, curation and information. An electronic database of the bird nest record cards was established in the late 1990s. The database was lost due to a computer fault but fortunately, the original nest record cards survived (although the rate of record submission has drastically declined and no electronic database was in place prior to this initiative). The entire nest record collection consists of some 16,600 cards out of which there are 3,200 waterbird records and with about 441 records for ducks. Time period: From 1920 to present | Nickson Otieno  National Museums of Kenya, Ornithology Section P. O. Box 40658-00100 Nairobi, Kenya. [neotieno@yahoo.com](mailto:neotieno@yahoo.com) |
| Namibia | Scientific Services, Ministry of Environment and Tourism Nest Record scheme, | Housed within the Avifaunal data base at Environmental Information Service Namibia  [www.the-eis.com](http://www.the-eis.com) | Historical nest records (e.g. CJ Andersson (1800s) ) to present. Started by Charles Clinning (1979), updated continuously by Chris Brown (1988-2000), Rob Simmons (2000-2013) and Alice Jarvis (present). 7,312 records to 2001, ongoing. Only about 200 records for ducks. | Alice Jarvis/ Tony Robertson  EIS webmasters, Windhoek, Namibia  [tr_aj@mweb.com.na](mailto:tr_aj@mweb.com.na) |
| South Africa | SAOS (South African Ornithological Society) nest cards, SABAP (South African Bird Atlas) 1, SABAP2, Harrismith collection, BirdLife South Africa nest cards, Rocher Pan data set (Cape Nature: Kevin Shaw/Cassie Heyl), additional personal records | Percy FitzPatrick Institute of African Ornithology, University of Cape Town (Niven Library); Animal Demography Unit (ADU), University of Cape Town. | Nest cards were first completed in South Africa under the auspices of the South African Ornithological Society (SAOS), which was later subsumed into BirdLife South Africa. Although the number of nest cards returned has declined in recent years, the number of bird breeding records obtained was boosted by records from the first and second southern African bird atlases (SABAP1 and SABAP2), which are stored in digital form only by the ADU. Collections include around 200,000 records in total, of which an estimated 80,000 are for waterbirds and approximately 20,000 for ducks. Time period is from 1900 to present. | PFIAO: cards are stored in the Niven Library at the University of Cape Town and curated by the Niven librarian.  For digital copies, contacts are:  SAOS cards: Graeme Cumming, gscumming@gmail.com;  ADU datasets: Animal Demography Unit, UCT |
| Tanzania | Tanzania bird atlas, additional records from Nairobi Museum holdings (East Africa Nest Record Card Scheme) | Iringa, Tanzania | The atlas data set is primarily distributional, but nest records are also documented for many species. | Neil and Liz Baker, Tanzania Bird Atlas, P.O. Box 1605, Iringa, Tanzania. tzbirdatlas@yahoo.co.uk |
| Zambia | BirdLife Zambia nest card record scheme, additional records from SAOS data set | Lusaka, Zambia | The dataset is relatively small (c. 2,000 cards in total) but exists in spreadsheet form. | BirdWatch Zambia, CEO Moses Nyoni http://www.birdwatchzambia.org/content/about.shtml |
| Zimbabwe | Birdlife Zimbabwe Nest Record Card Scheme, GRIPAVI-NUST project, and SABAP 1. | Ornithology Department, Natural History Museum, Bulawayo | Nest Record Cards were first completed in the then Rhodesia under the guidance of the Rhodesian Ornithological Society (ROS). The project continued post-independence under Ornithological Association of Zimbabwe (OAZ). The OAZ was subsumed into Birdlife Zimbabwe in 2002 when they became a full partner of Birdlife International. Although most of the Nest Record Cards are collected by Birdlife Zimbabwe members, they are stored in the Ornithology department of the Natural History Museum in Bulawayo. The Museum in conjunction with Birdlife Zimbabwe has started digitizing all nest record cards. The number of Nest Record Cards returned to the Natural History Museum has generally been in decline post year 2000.  The entire nest record scheme collection includes around 40 000 records of which an estimated 5000 are for waterbirds and 1126 are for ducks. The records are from 1910 to date. | NUST:Josphine Mundava, [jmundava@gmail.com](mailto:jmundava@gmail.com)  Julia Dupree, Birdlife Zimbabwe/Natural History Museum, Ornithology Department [daliadupree@gmail.com](mailto:daliadupree@gmail.com) |
