# Supplementary material for: Timing and location of reproduction in African waterfowl: an overview of >100 years of nest records
Source: Ecol Evol. 2016 Jan 18;6(3):631–46. doi: 10.1002/ece3.1853 (PMC4739573; doi:10.1002/ece3.1853)
Supplement: Supplementary file 2 — Appendix S2. Data capture considerations. This appendix contains a summary of the approach that we used in data capture to ensure consistency in data capture, coordinates, and estimates of hatching date. [file ECE3-6-631-s002.docx]

**Appendix 1: Methods used in capturing data from nest record cards**

After capturing all available data in spreadsheet format, we (1) standardized nesting dates to an estimated hatching date; and (2) georeferenced each record.

*1. Standardization to hatching date*

Hatching dates were estimated according to the following guidelines. We assigned each record a confidence level (1=high, 3=low) to permit people using the data to filter out lower quality data where necessary.

Table A1.1: Guidelines for interpreting descriptions of breeding observations given on nest cards.

**Guideline:**

**Description given Assumption Method Confidence**

**(date of hatching)**

“just hatched” < 3 days subtract ±3 days 1

“newly hatched” < 7 days subtract ± 5 days 1

“tiny” < 7 days subtract ±7 days 1

“very small” < 10 days subtract ± 10 days 1

“small” ± 10 days subtract ± 10 days 1

“downy” “fluffy” < 2 weeks subtract ± 10 days 1

estimate provided e.g. “5 days old” subtract to estimated age 1

“fledged” reached fledging age & species dependent 1

able to fly

“chick” ≤ 2 weeks subtract ± 2 weeks 2

“duckling” not yet juvenile subtract 2 to 3 ½ weeks 2

(species dependent)

“juvenile” older than half grown subtract ≥ half grown age 2

(species dependent)

“nesting”/”breeding” about to incubate Add 2-3 days for egg-laying & 2

length of incubation period

(species dependent)

“eggs” seen mid-way through Add half length of incubation 2

incubation period period (species dependent)

poor description e.g. “young with parents” copy date across 3

no description - leave blank 4

1=accurate within 1 week

2=accurate 2-4 weeks

3=>4 weeks

4=unknown

Table A1.1 offers a guideline for how hatching dates were estimated in most instances. In some cases, more detailed descriptions of chicks were provided and hatching dates were then estimated accordingly. For instance, it may have been described as a “chick” or “juvenile”, but the description may have given another clue relating to physiology or behaviour which would have influenced the hatching date estimation (e.g. stripy, sub-adult plumage).

*2. Georeferencing*

Georeferencing of observations was undertaken in several different ways, depending on the data provided:

1. Observations with observer-assigned coordinates – these were used as given except in cases where there were obvious errors that demanded reassignment of locations.
2. Observations with associated grid references from survey maps (including regular survey maps and quarter-degree cells from Bird Atlasing projects) – we used the letter-number combination of survey maps to determine a central coordinate for each cell and assigned the record to the centre of the cell.
3. Observations with place name provided – these were individually tracked down, either by asking the data provider or using Google Earth. When using Google Earth, coordinates were assigned to the nearest shore of the most likely wetland at or near to the location. For example, a record indicating “Norton” in Zimbabwe would be assigned to the nearby shoreline of Lake Chivero. In some cases, records without coordinates were from birding sites known to the authors, and in these instances, we drew on our own knowledge of the site to pinpoint likely nesting locations.

The quality of each data point (or more correctly, our confidence in it) was assigned a code that would enable the user to strip away poorer-quality coordinates. Codes were assigned according to the likely order of magnitude of potential error. GPS-derived coordinates, with errors up to tens of metres, were given rating “1” (highest quality). Errors up to hundreds of metres were given code 2; of up to 10 kilometres, code 3; and of potentially up to 100 kilometres (although typically expected to be less), code 4. For the analysis, we used data up to the level of code 3.

All coordinates given in the database are in decimal degrees in a lat-long projection, using the WGS 80 spheroid datum.
