# Supplementary figures and images for: Timing and location of reproduction in African waterfowl: an overview of >100 years of nest records
Source: Ecol Evol. 2016 Jan 18;6(3):631–46. doi: 10.1002/ece3.1853 (PMC4739573; doi:10.1002/ece3.1853)

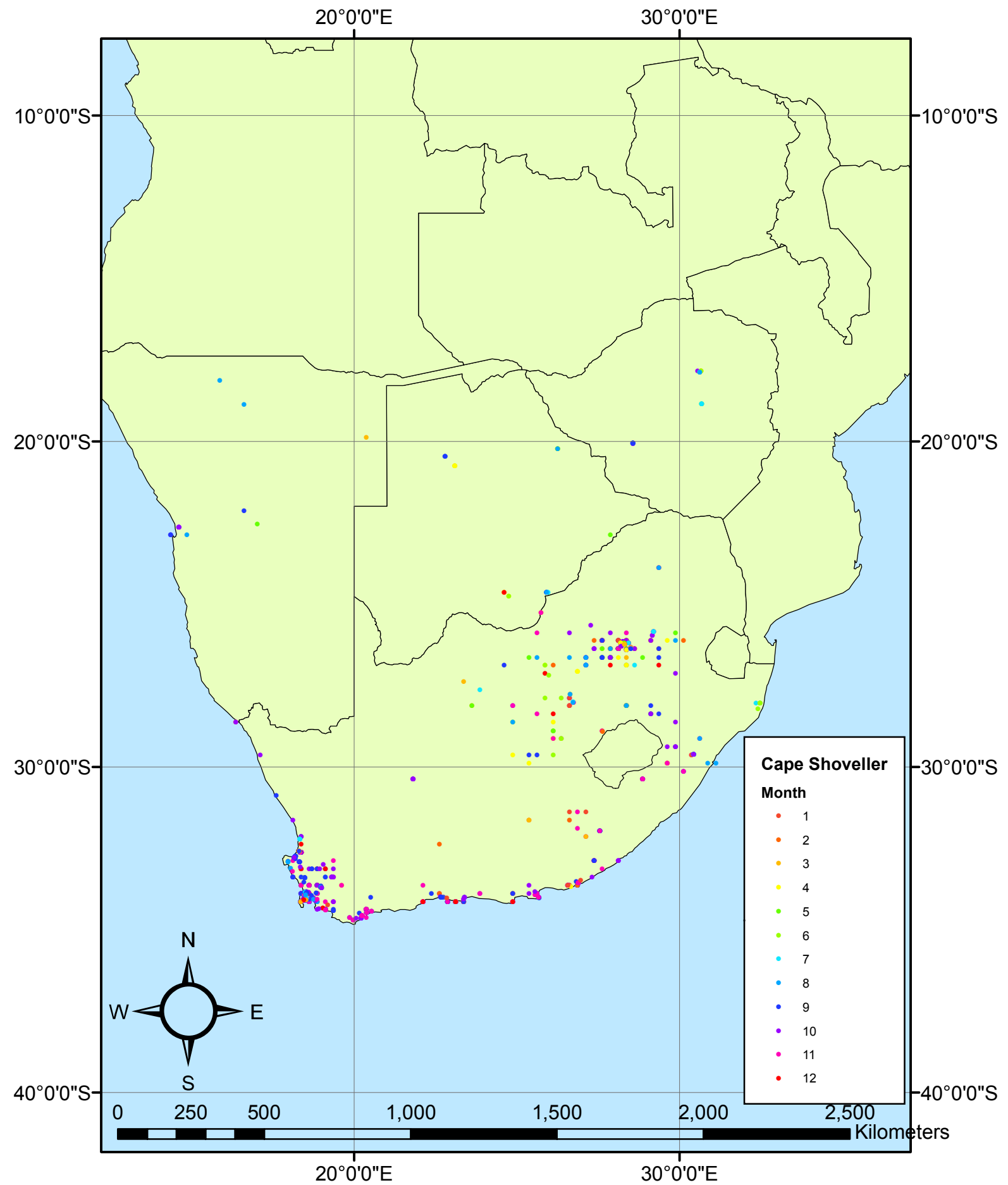

Supplement: Supplementary file 4 — Appendix S4. Distribution maps of breeding data for all Afrotropical species considered in the analysis. [file ECE3-6-631-s004.pdf]

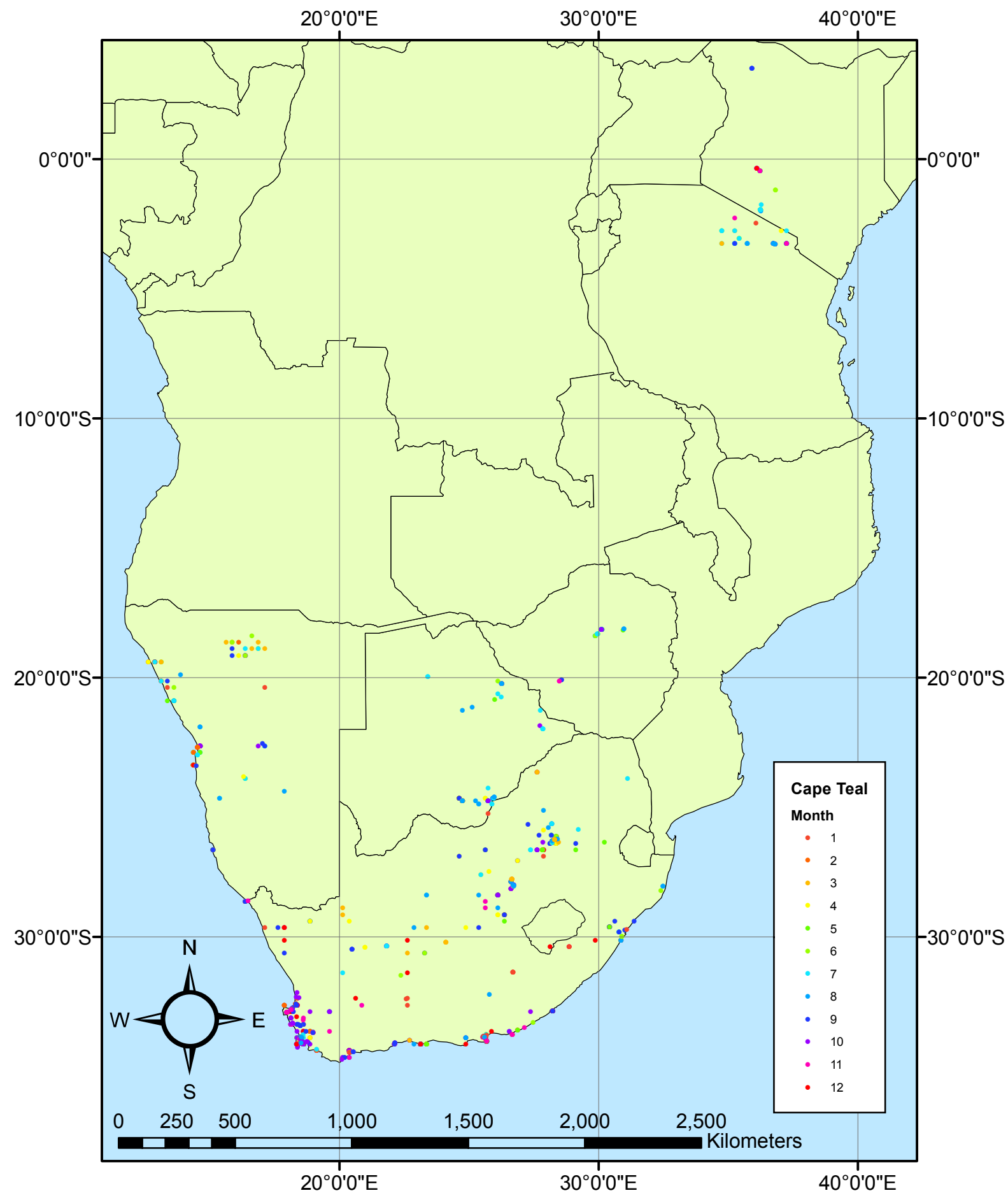

Supplement: Supplementary file 5 [file ECE3-6-631-s005.pdf]

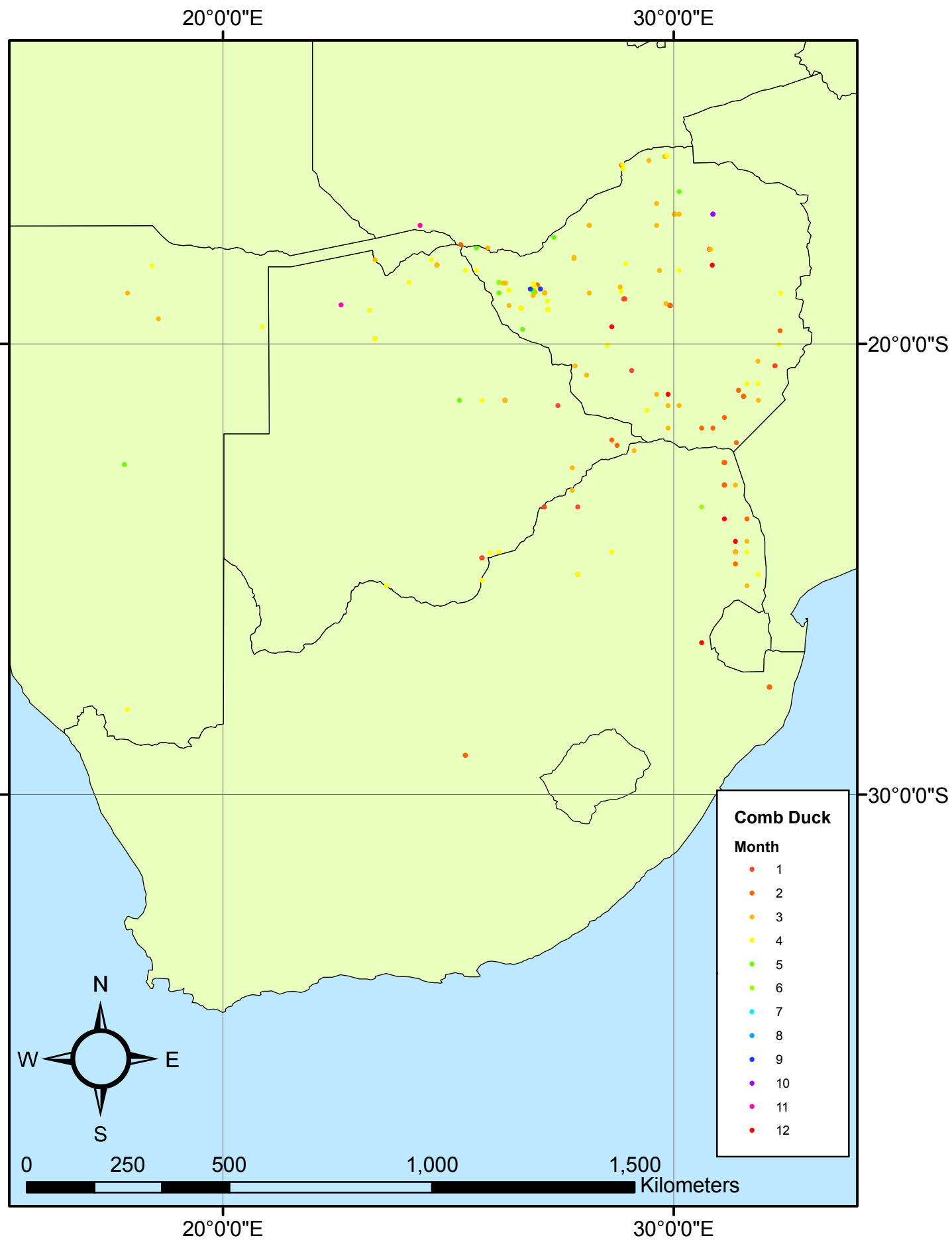

Supplement: Supplementary file 6 [file ECE3-6-631-s006.pdf]

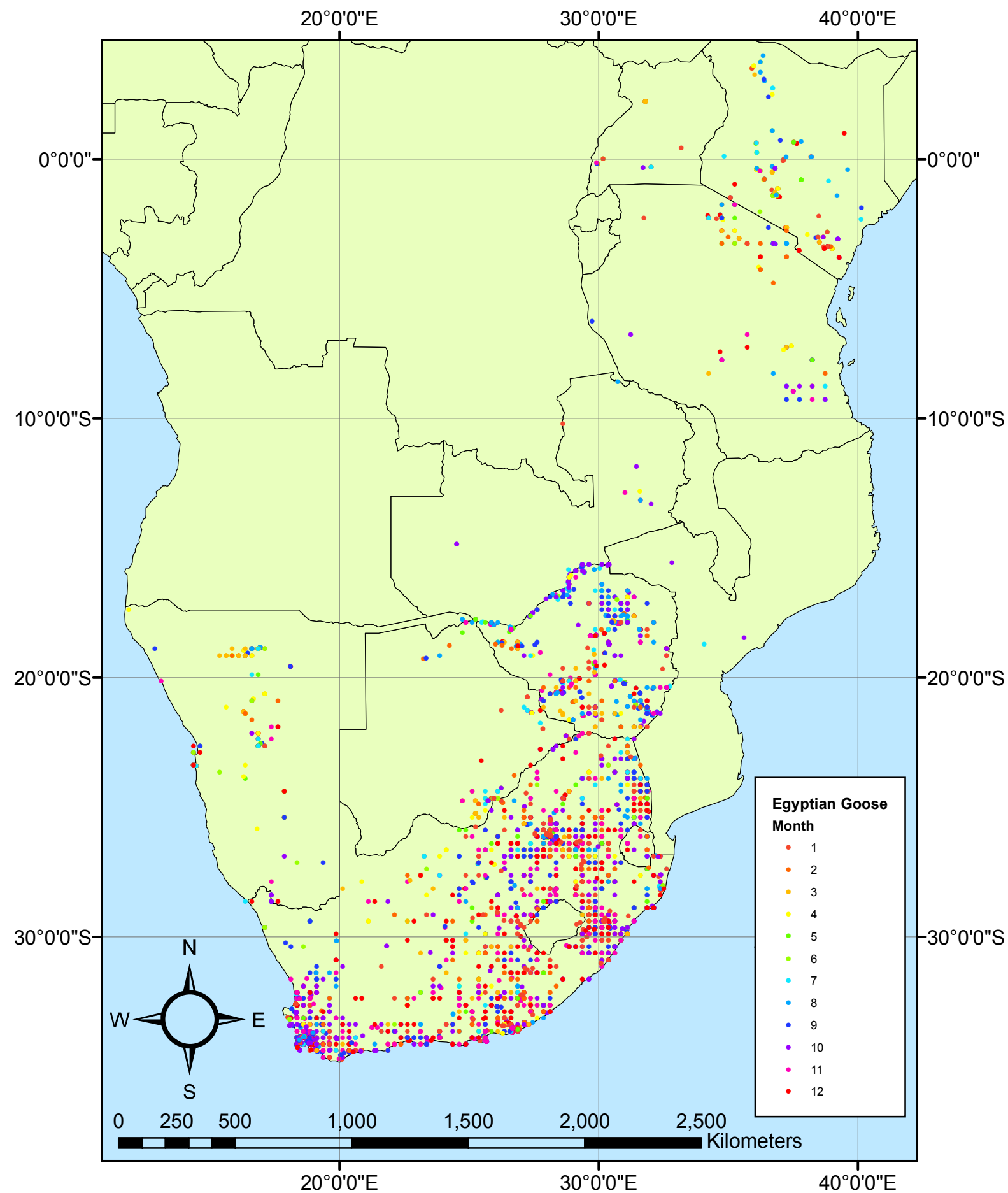

Supplement: Supplementary file 7 [file ECE3-6-631-s007.pdf]

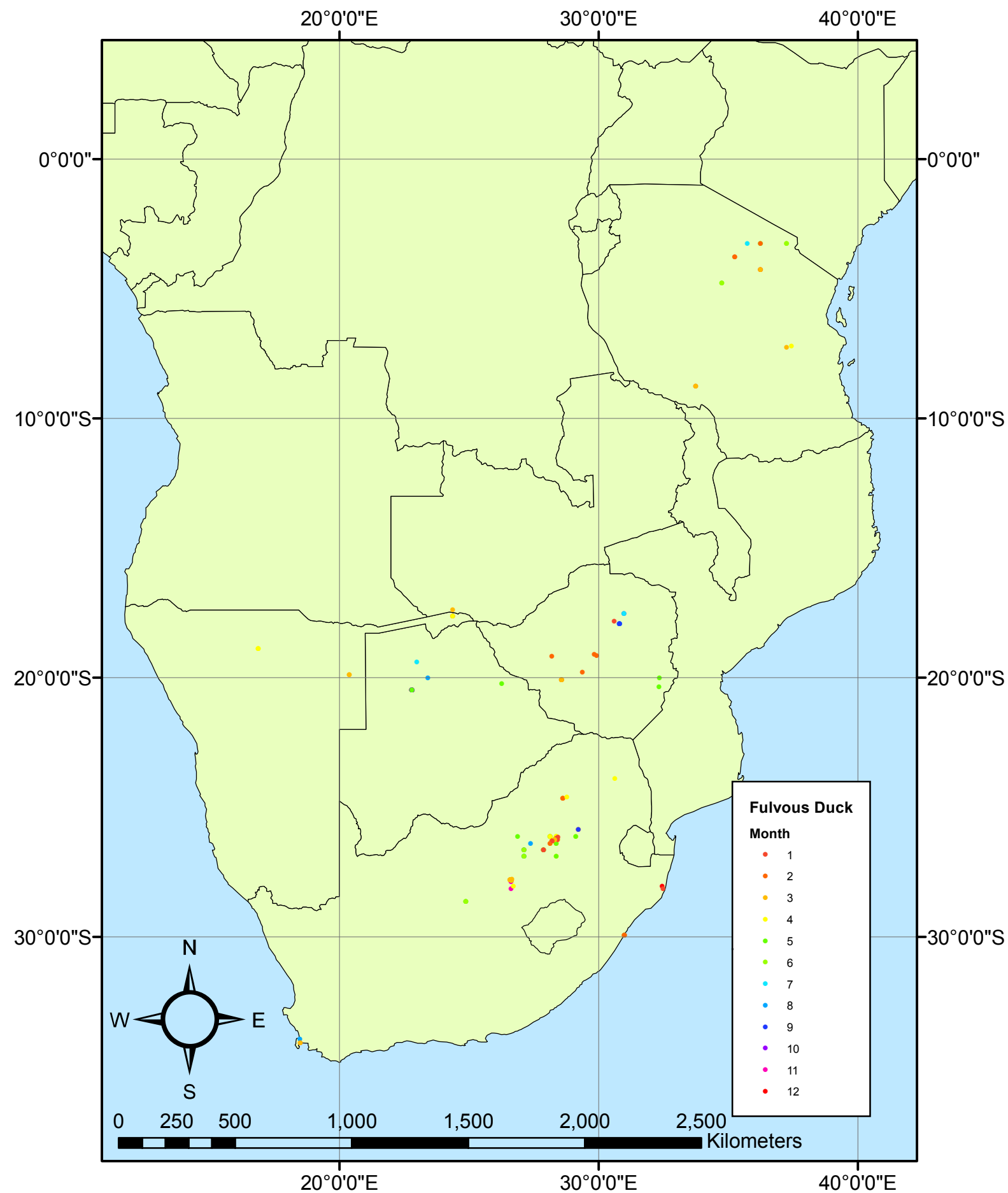

Supplement: Supplementary file 8 [file ECE3-6-631-s008.pdf]

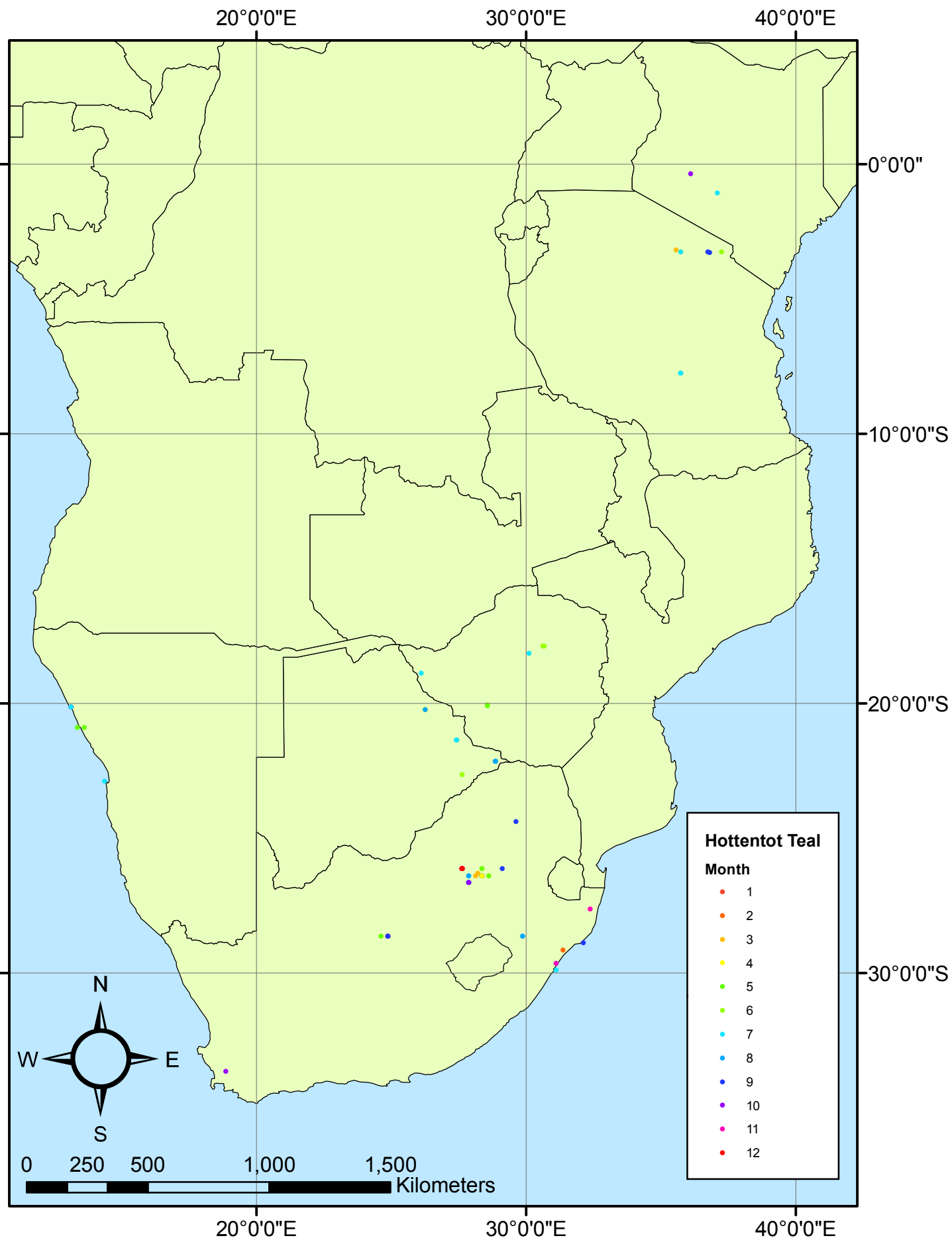

Supplement: Supplementary file 9 [file ECE3-6-631-s009.pdf]

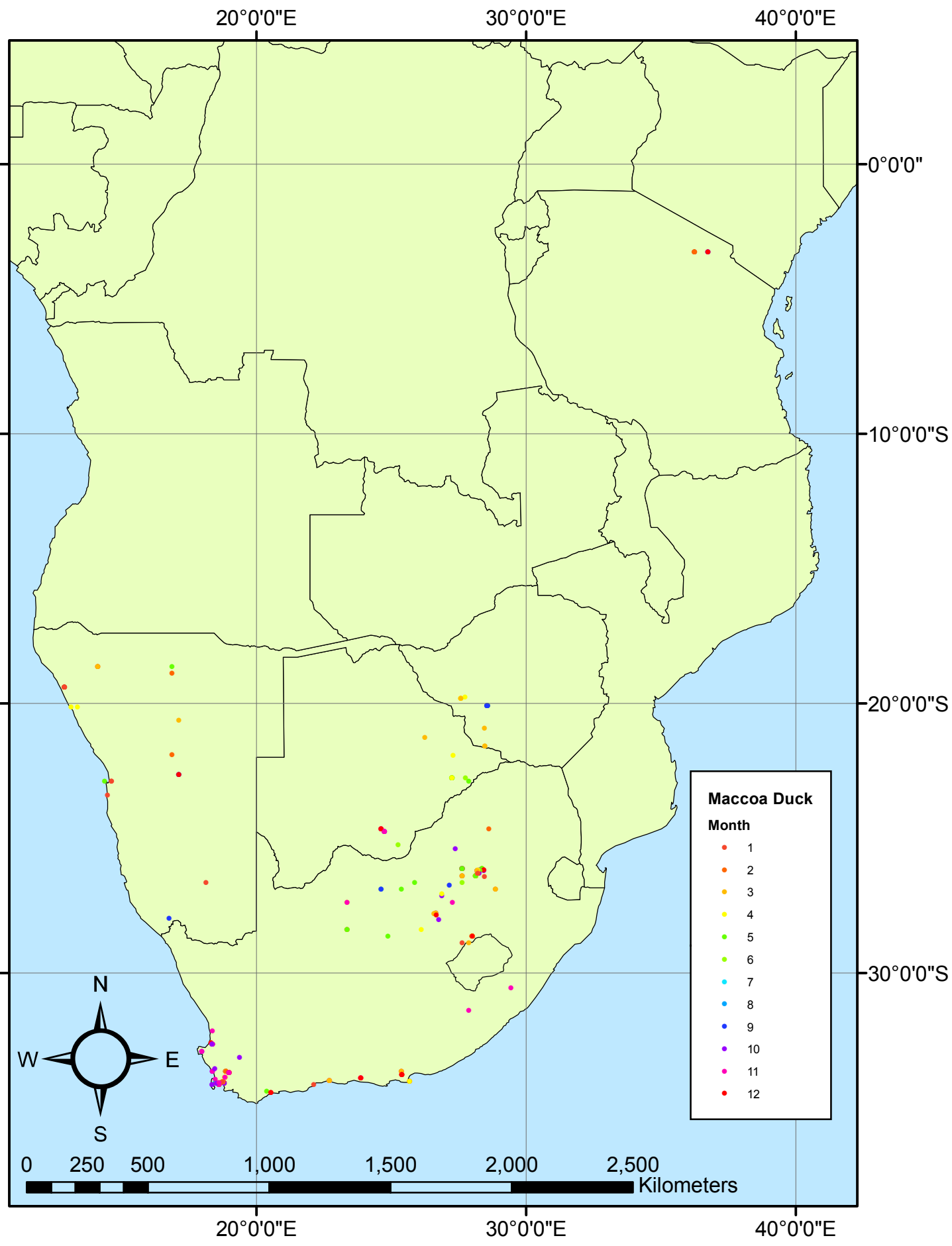

Supplement: Supplementary file 10 [file ECE3-6-631-s010.pdf]

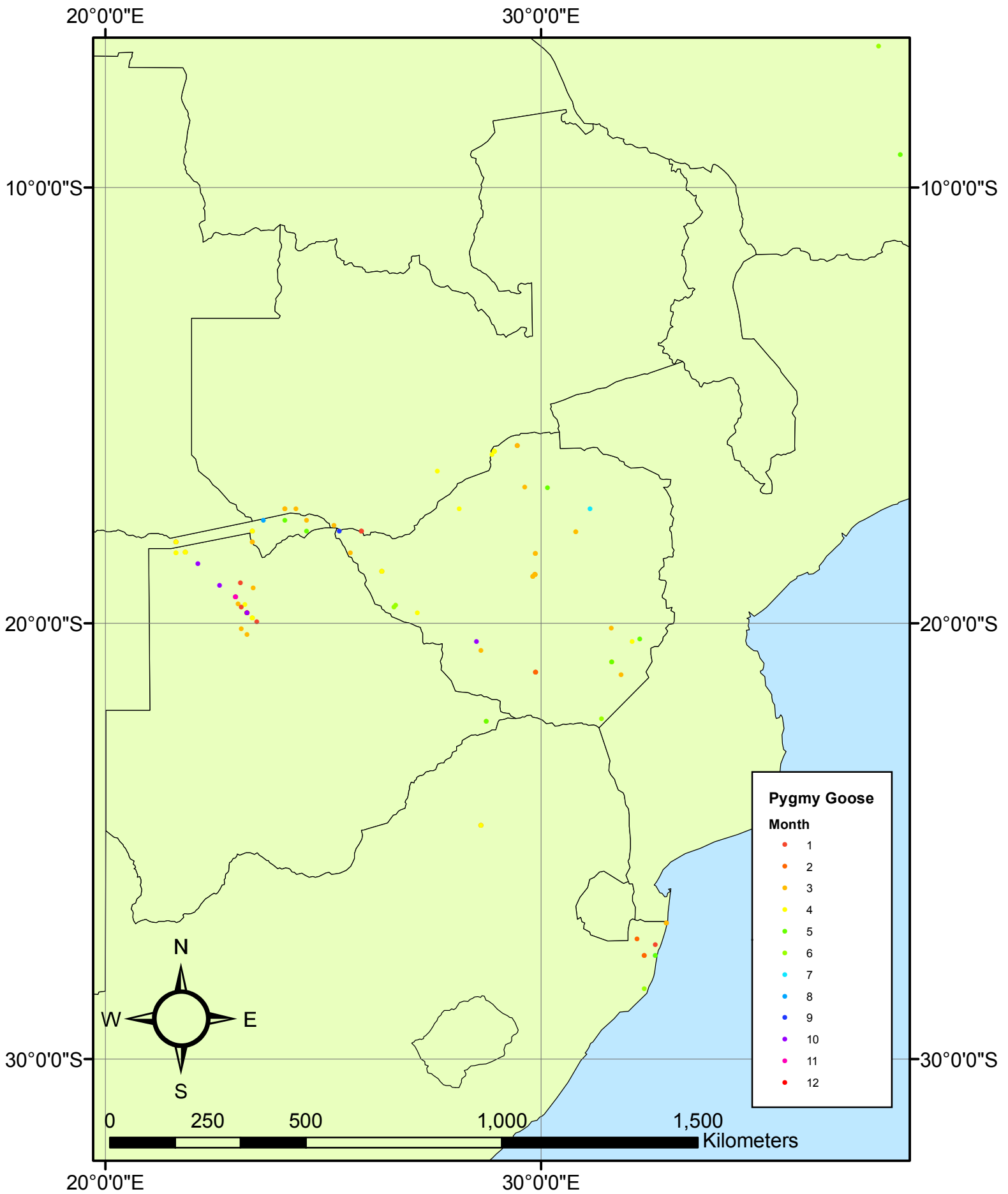

Supplement: Supplementary file 11 [file ECE3-6-631-s011.pdf]

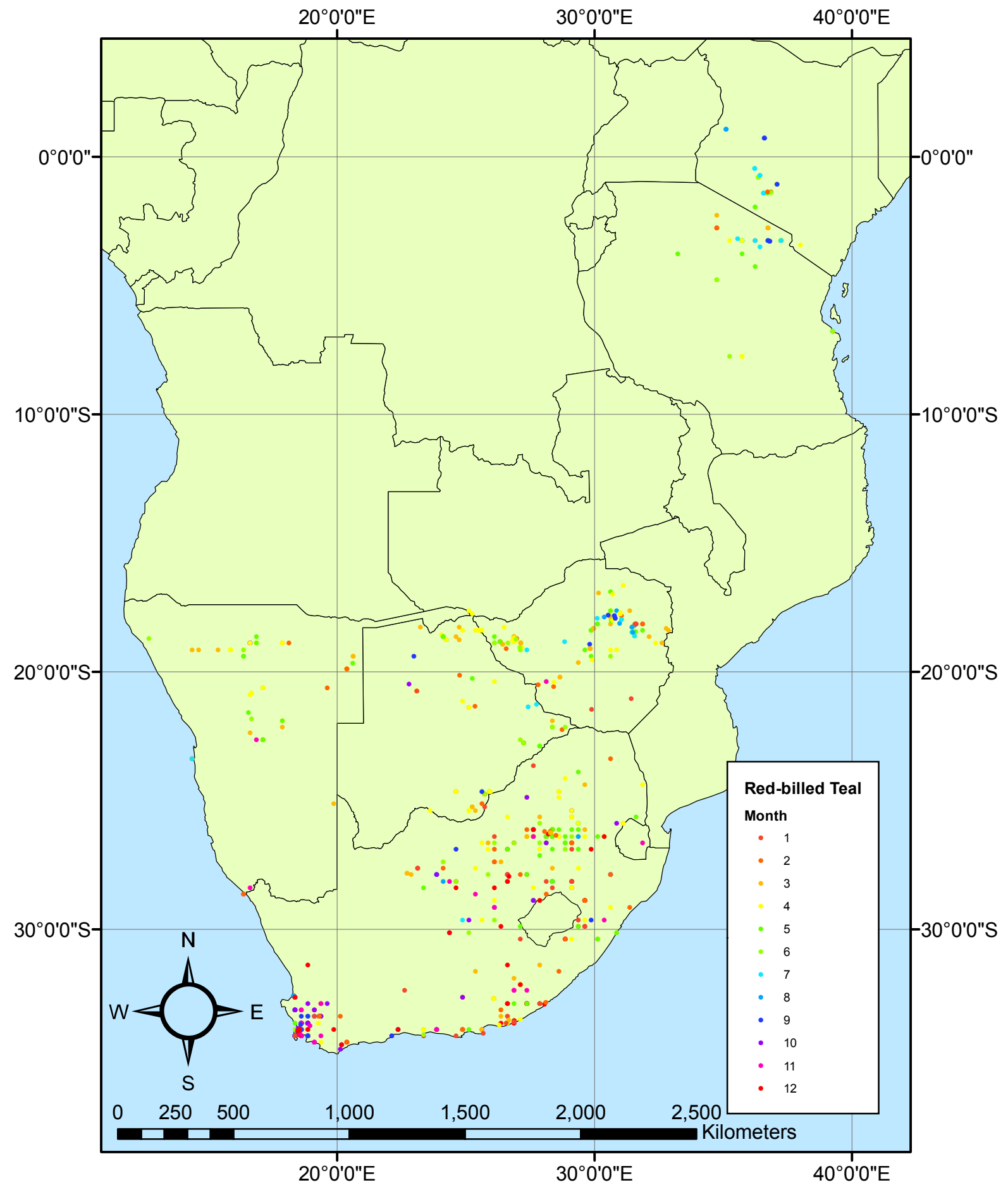

Supplement: Supplementary file 12 [file ECE3-6-631-s012.pdf]

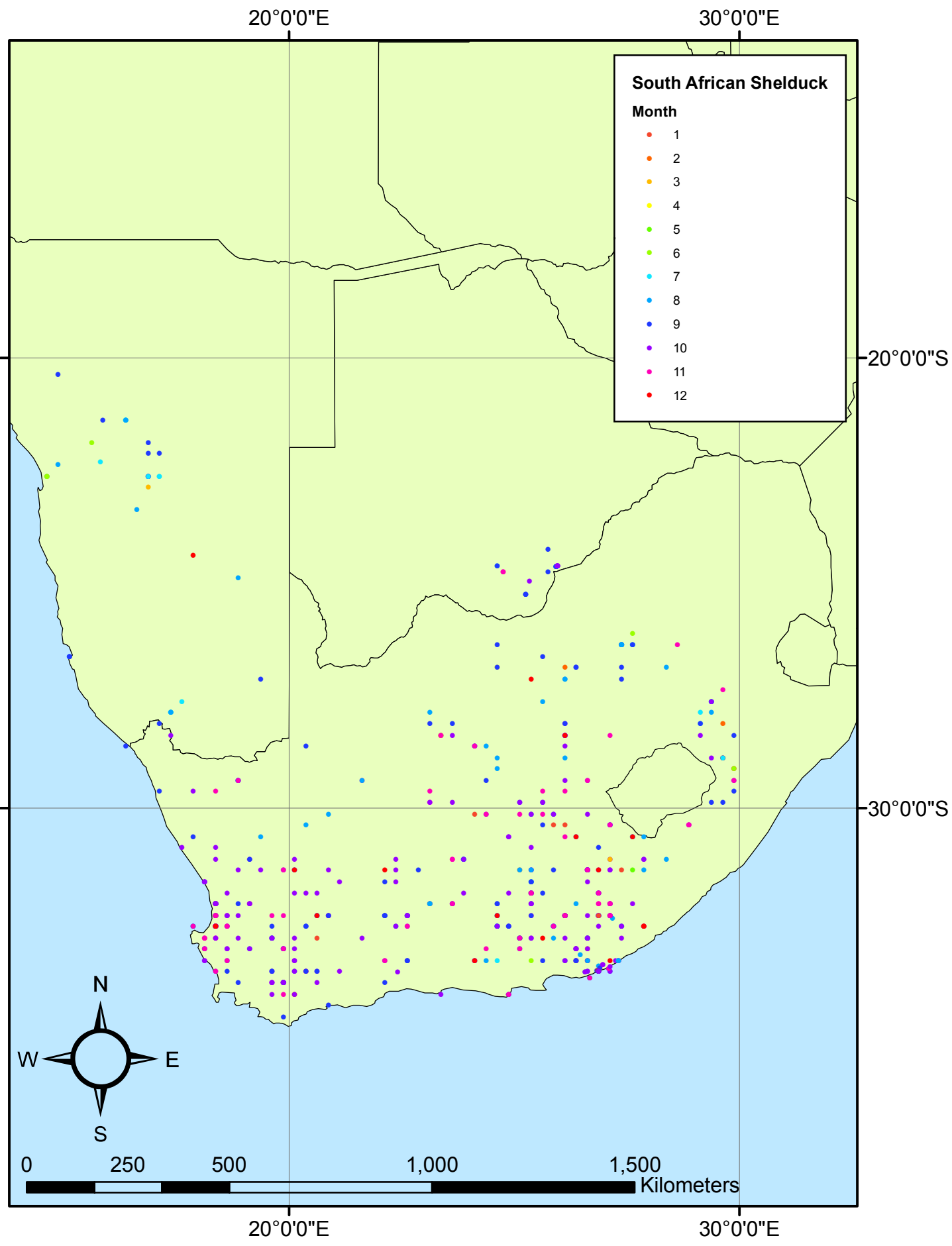

Supplement: Supplementary file 13 [file ECE3-6-631-s013.pdf]

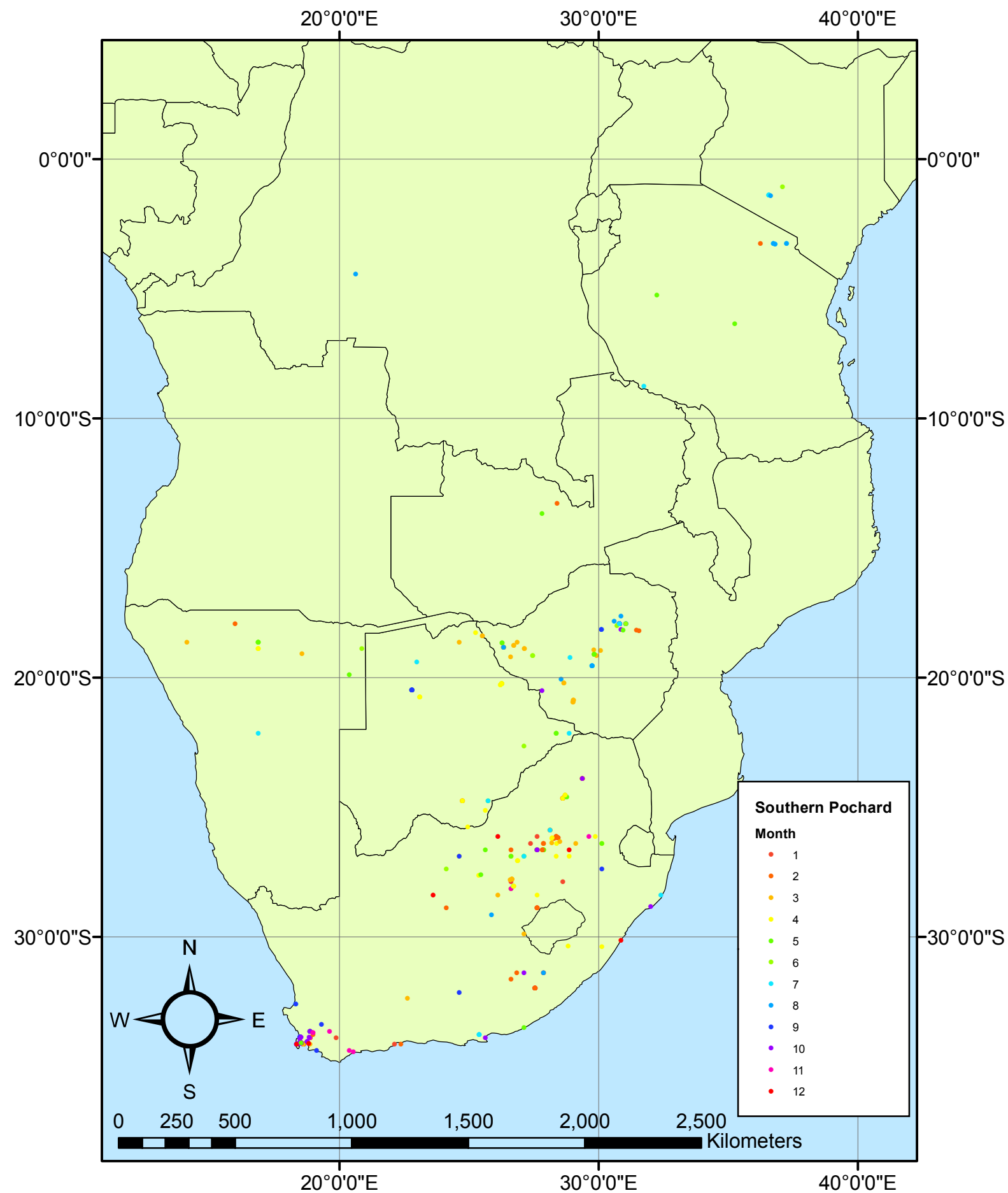

Supplement: Supplementary file 14 [file ECE3-6-631-s014.pdf]

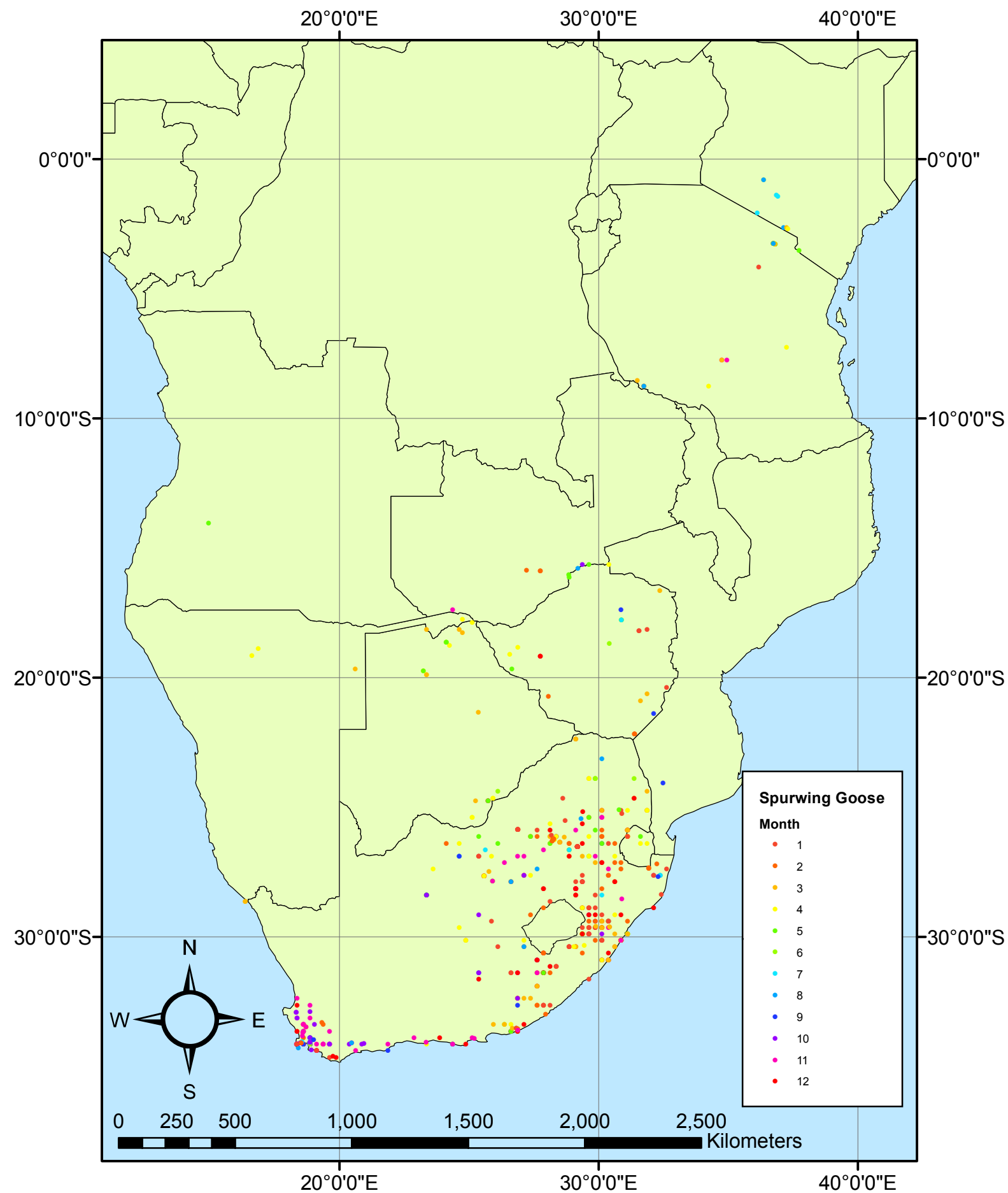

Supplement: Supplementary file 15 [file ECE3-6-631-s015.pdf]

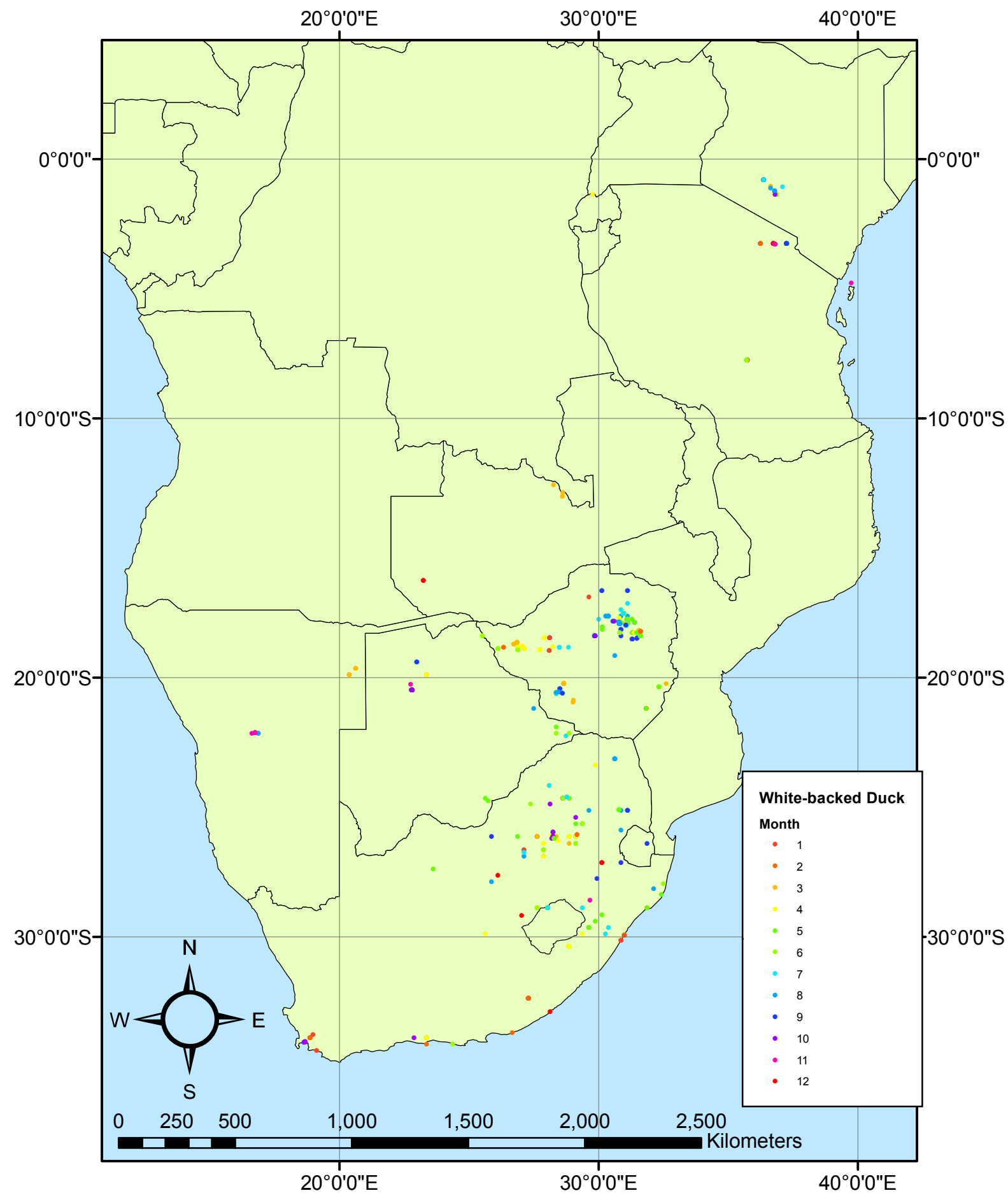

Supplement: Supplementary file 16 [file ECE3-6-631-s016.pdf]

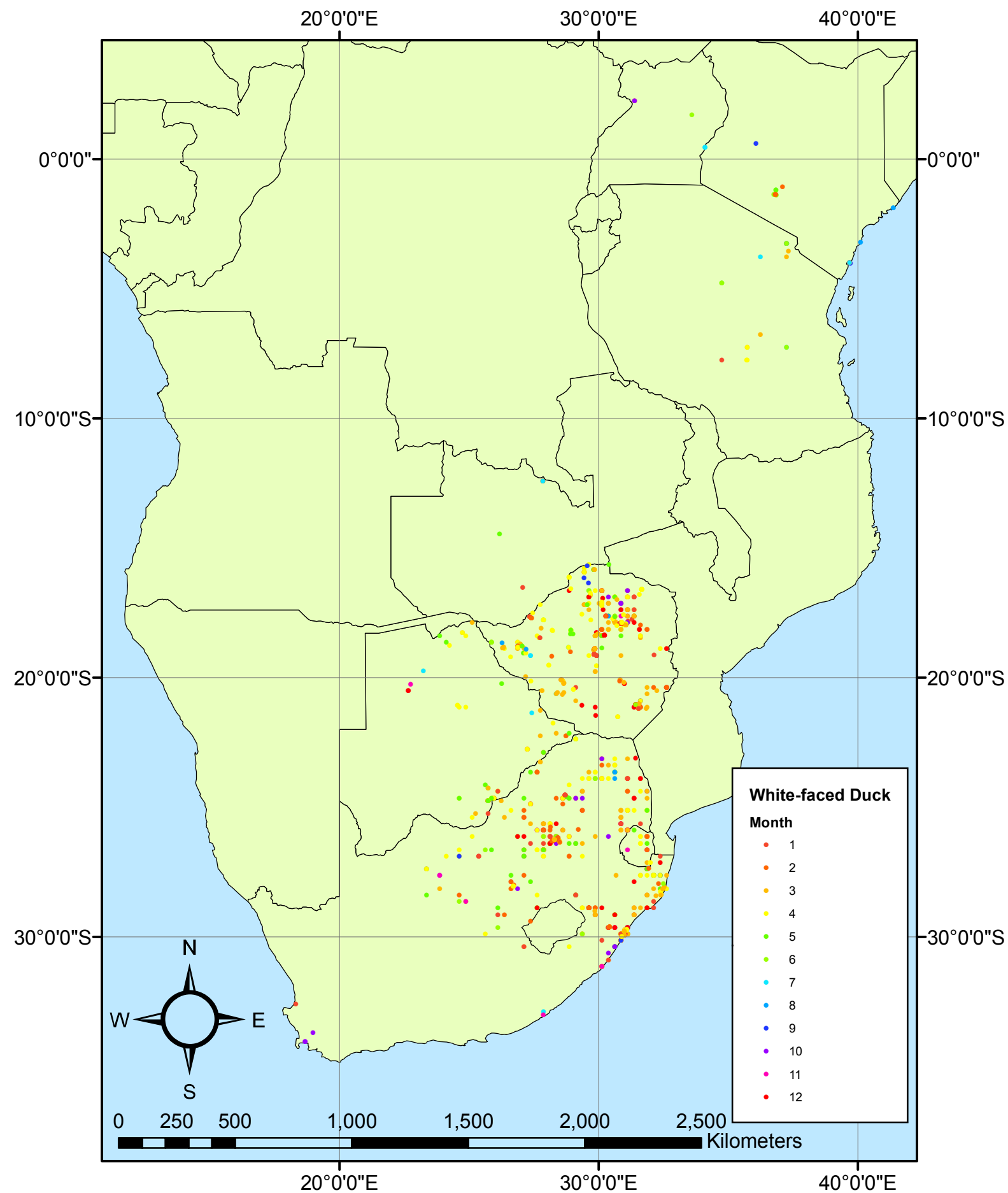

Supplement: Supplementary file 17 [file ECE3-6-631-s017.pdf]

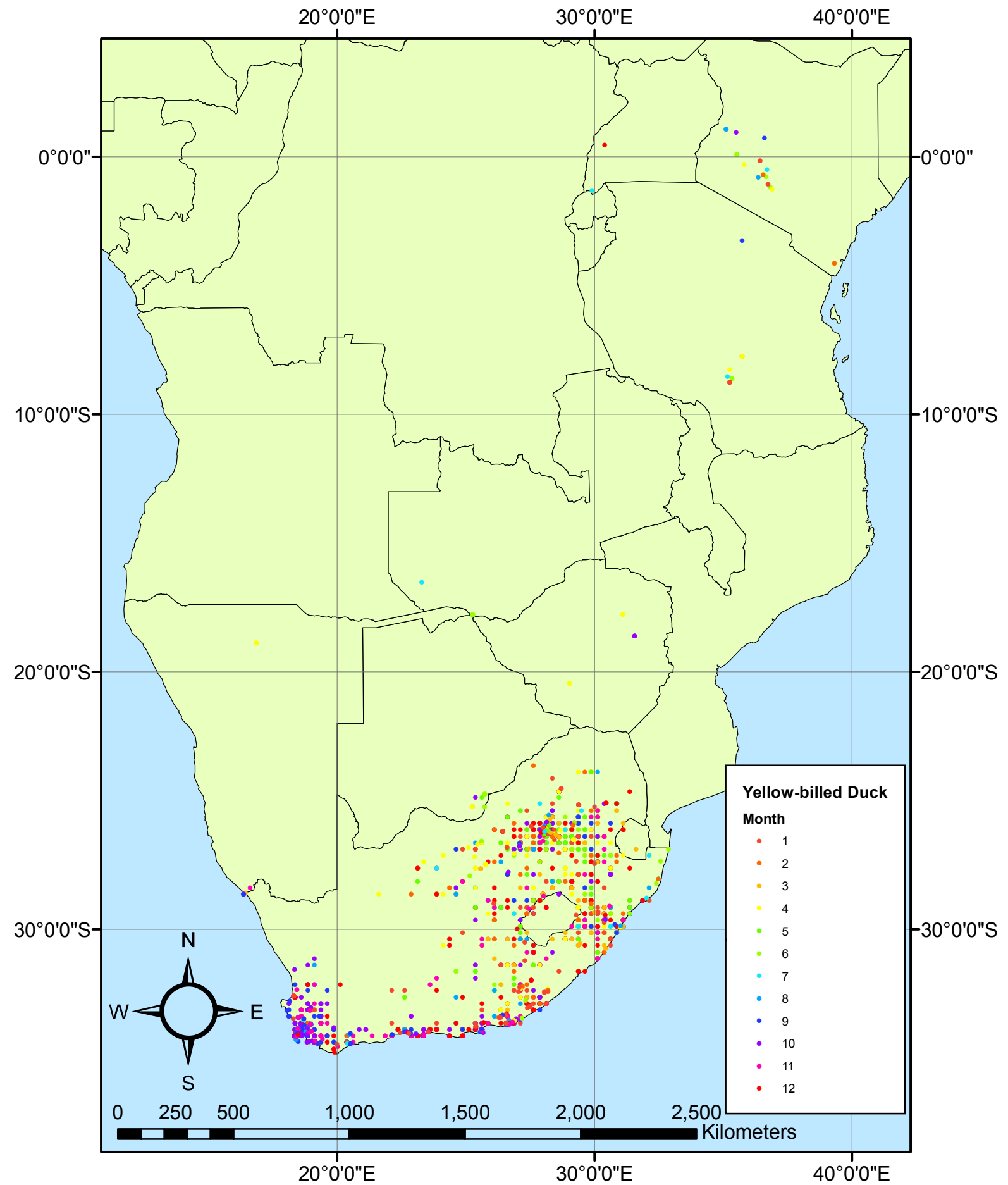

Supplement: Supplementary file 18 [file ECE3-6-631-s018.pdf]

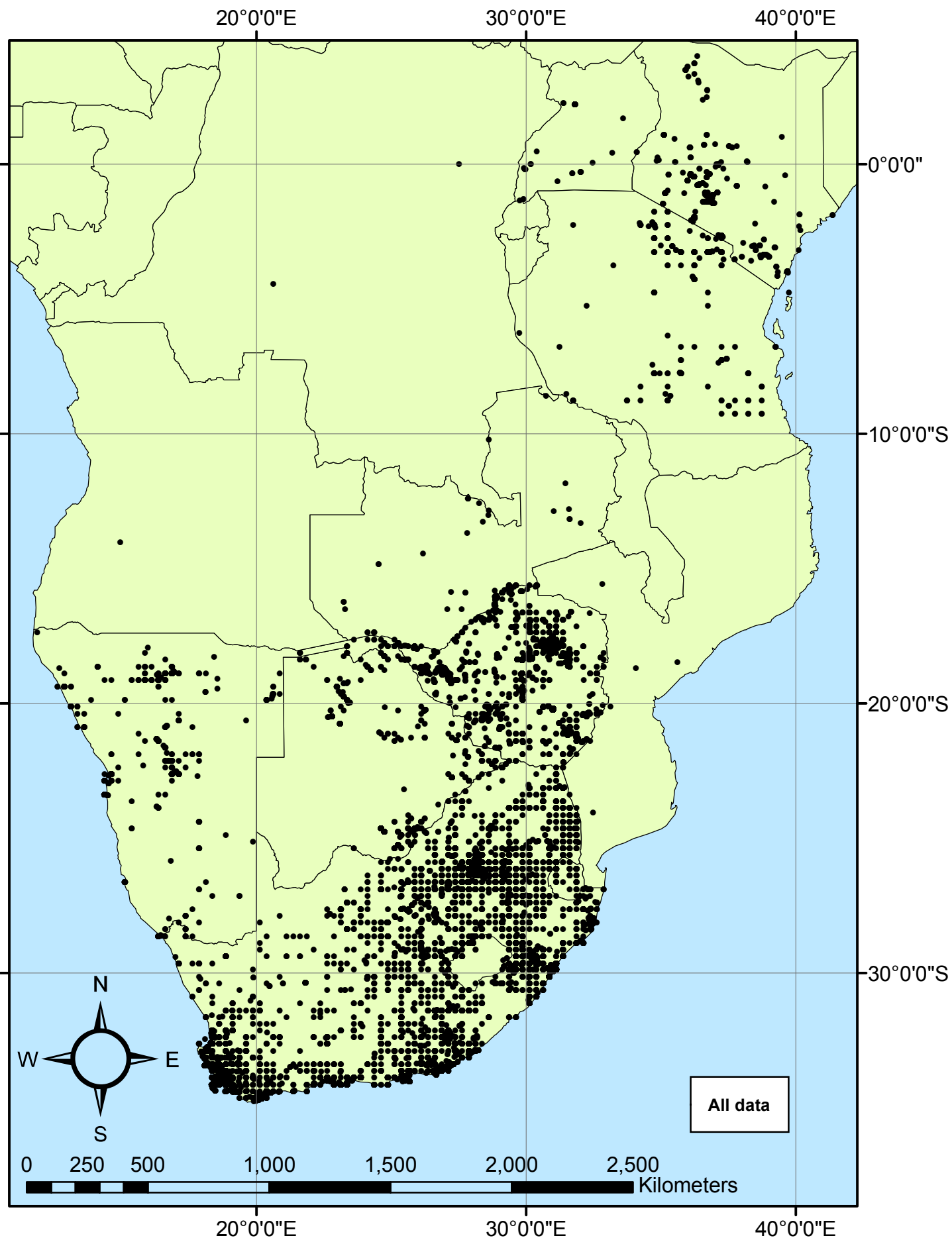

Supplement: Supplementary file 19 [file ECE3-6-631-s019.pdf]

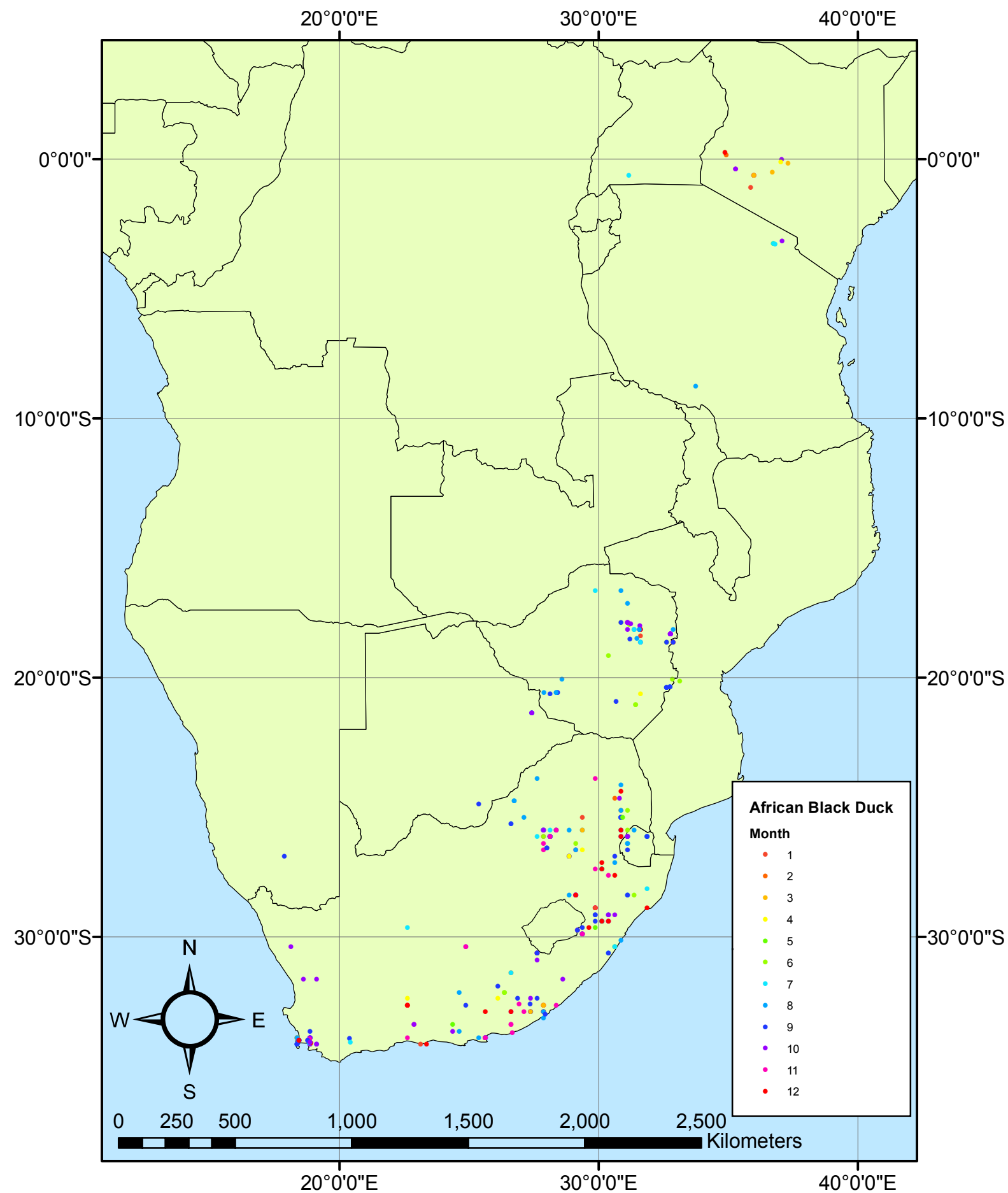

Supplement: Supplementary file 20 [file ECE3-6-631-s020.pdf]
